# Enhancement of pyocyanin production by subinhibitory concentration of royal jelly in *Pseudomonas aeruginosa*

**DOI:** 10.12688/f1000research.27915.1

**Published:** 2021-01-11

**Authors:** Dina Auliya Amly, Puspita Hajardhini, Alma Linggar Jonarta, Heribertus Dedy Kusuma Yulianto, Heni Susilowati

**Affiliations:** 1 Master of Dental Sciences Program, Faculty of Dentistry, Universitas Gadjah Mada, Sleman, Yogyakarta, 55281, Indonesia; 2 Department of Oral Biology, Faculty of Dentistry, Universitas Gadjah Mada, Sleman, Yogyakarta, 55281, Indonesia; 3 Department of Dental Biomedical Sciences, Faculty of Dentistry, Universitas Gadjah Mada, Sleman, Yogyakarta, 55281, Indonesia

**Keywords:** royal jelly, antibacterial effect, Pseudomonas aeruginosa, pyocyanin

## Abstract

**Background: ***Pseudomonas aeruginosa*, a multidrug resistant Gram-negative bacterium, produces pyocyanin, a virulence factor associated with antibiotic tolerance. High concentrations of royal jelly have an antibacterial effect, which may have the potential to overcome antibacterial resistance. However, in some cases, antibiotic tolerance can occur due to prolonged stress of low-dose antibacterial agents. This study aimed to investigate the effect of subinhibitory concentrations of royal jelly on bacterial growth and pyocyanin production of *P. aeruginosa*.

**Methods:***Pseudomonas aeruginosa* ATCC ^® ^10145™ and clinical isolates were cultured *in* **BHI media for 18 hours followed by optical density measurements at 600 nm wavelength to determine minimum inhibitory concentration (MIC). After 36 hours of incubation, pyocyanin production was observed by measuring the absorbance at 690 nm. Pyocyanin concentrations were calculated using extinction coefficient 4310 M ^-1^cm ^-1^.

**Results: **Results of the MIC tests of both strains were 25%. The highest production of pyocyanin was observed in the subinhibitory concentration group 6.25%, which gradually decreased along with the decrease of royal jelly concentration. Results of one-way ANOVA tests differed significantly in pyocyanin production of the two strains between the royal jelly groups. Tukey HSD test showed concentrations of 12.5%, 6.25%, and 3.125% significantly increased pyocyanin production of ATCC ^® ^10145™, and the concentrations of 12.5% and 6.25% significantly increased production of the clinical isolates.

**Conclusions: **This study concluded royal jelly concentrations of 25% or above could inhibit bacterial growth; however, only the concentrations of 12.5% and 6.25% could increase pyocyanin production in *P. aeruginosa, *both in ATCC ^®^ 10145™ and clinical isolates. In conclusion, it is advisable to determine the appropriate concentration of royal jelly to obtain beneficial virulence inhibiting activity.

## Introduction

*Pseudomonas aeruginosa* ( *P. aeruginosa*) is one of the Gram-negative bacilli bacteria which causes nosocomial infections that can be fatal, especially in immunocompromised patients ^1– 3^. These bacteria are often found in the dental unit waterlines which allows the transmission of these bacteria into the oral cavity ^4^. As an opportunist pathogen, *P. aeruginosa* is also frequently involved in oral infections, such as necrotizing ulcerative gingivitis, periodontitis, and mandibular osteomyelitis ^5– 7^. Although the mechanism is not clear yet, its presence in the oral cavity has been shown to result in systemic infections, such as nosocomial pneumonia ^8^.

Based on the reports from several clinical cases, the infection caused by *P. aeruginosa* bacteria can be fatal. Treatment of *P. aeruginosa* infection is sometimes ineffective, which is closely related to the number of virulence factors possessed by the bacteria ^9^. The bacterial cell surface components and some secretory products are important virulence factors of *P. aeruginosa*, one of which is pyocyanin ^10^. Pyocyanin is a cytotoxic pigment from the Phenazine group of compounds that can facilitate biofilm development, cause pro-inflammatory effects, and result in host cell death ^11^.

The resistance of *P. aeruginosa* to various spectrums of antibiotics creates difficulties in handling the infection it causes ^12^. It has been reported recently that the administration of antibiotics below the minimum inhibitory concentration (MIC) can cause specific bacterial responses, such as an increase in pyocyanin production in *P. aeruginosa*. PAO1 and P14 are the attempts by the bacteria to survive under antibiotic stress ^13^. This certainly motivates researchers to further analyze the infection they cause, and find the appropriate antibiotic concentration or dose to overcome the problem.

Royal jelly is a natural bee product that has the potential to be developed to overcome antibiotic resistance. Royal jelly has anti-inflammatory, antibacterial, and antioxidant effects ^14^. Royal jelly proteins, such as Jelleine, major royal jelly protein-1 (MRJP1), and royalicin are known to have antibacterial effects against *P. aeruginosa*. Major royal jelly protein-1 and Jelleine can interfere with the permeability of the outer membrane of the cell, causing the loss of vital contents of bacterial cells, which in turn causes cell death. Cationic antimicrobial peptides, such as royalicin, are known to also interfere with cell membrane permeability in various Gram-positive and Gram-negative bacteria, such as *P. aeruginosa* ^15– 17^. Results of previous studies have shown that royal jelly can inhibit the growth of *P. aeruginosa*. In this study, royal jelly showed inhibition of the growth of *P. aeruginosa* ATCC ^®^ 27853™ ^18^. In addition, it has also been known that royal jelly in various concentrations can inhibit the nonspecific attachment of *P. aeruginosa* ATCC ^®^ 27853™ ^19^, but so far, the effect of the subinhibitory concentration of royal jelly against these bacteria is unknown. Furthermore, as pyocyanin is an indicator of the pathogenicity of *P. aeruginosa* strains, the aim of this study was to determine the effect of subinhibitory royal jelly concentration on pyocyanin production in representative strains of a high level pyocyanin-producer *P. aeruginosa* ATCC ^®^ 10145™ and clinical isolates.

## Methods

This *in vitro* laboratory experimental research was done at the Integrated Research Laboratory of the Faculty of Dentistry, Universitas Gadjah Mada, Yogyakarta. All research procedures have been approved by the Ethics Committee of the Faculty of Dentistry, Universitas Gadjah Mada, Yogyakarta (No. 00393/KKEP/ FKG-UGM/EC/2020).

The royal jelly used in this study was obtained from Nusukan, Surakarta, Central Java, Indonesia. This product is produced from *Apis mellifera* bees that have been identified previously ^19^. Royal jelly 5.5 grams was dissolved in 10 ml of cold phosphate buffered saline (PBS), then homogenized using a magnetic stirrer (24 hours, 4°C). The royal jelly solution was centrifuged (12,000 g, 45 minutes, 4°C), then the supernatant was filtered using 0.45 µm millipore to produce 55% royal jelly. Furthermore, royal jelly was stored at a temperature of 4–8°C ^20^.

*Pseudomonas aeruginosa* ATCC ^®^ 10145™ (Thermo Scientific) was obtained from the Integrated Research Laboratory of the Faculty of Dentistry, Universitas Gadjah Mada. A clinical isolate of *P. aeruginosa* derived from patient sputum was obtained from the Laboratory of Microbiology, Faculty of Medicine, Public Health and Nursing, Universitas Gadjah Mada. Both of these strains were each inoculated in Luria Bertani broth and incubated at 37°C for 24 hours. After that, the culture was centrifuged at 3000 rpm for 15 minutes and then resuspended using 0.98% NaCl to obtain a bacterial concentration equivalent to 1.5 × 10 ^5^ CFU/ml.

### Measurement of the effect of royal jelly on the viability of *P. aeruginosa*

A sterile 55% w/v royal jelly solution was diluted in brain heart infusion (BHI; Himedia Laboratories) broth to obtain a concentration of 50% and then serial dilution was performed in 96 well microplates. A total of 5 µl of the *P. aeruginosa* ATCC ^®^ 10145™ suspension or clinical isolate bacteria (1.5 × 10 ^5^ CFU/ml) was inoculated in all groups, except the groups that had been determined as blanks (blanko). The culture was then incubated at 37°C for 18 hours. After that, the microplate was scanned using the Spark® Multimode Microplate Reader (Tecan trading AG) to measure optical density (OD) using a 600 nm wavelength. The percentage of bacterial viability inhibition was determined based on the OD value of the treatment group against the control.

### Analysis of the effect of royal jelly on pyocyanin production

Royal jelly solution was diluted into sterile BHI broth to get the concentration of 12.5%, 6.25%, 3.125%, 1.56%, 0.78%, 0.39%, 0.19%, and 0.098% w/v. Both strains of *P. aeruginosa* were cultured on BHI broth containing various concentrations of royal jelly as treatment and BHI broth only as a blank (blanko). The cultures were incubated at 37°C for 36 hours, then the pyocyanin production of each strain was observed visually, which appeared green in the culture supernatant. The pyocyanin concentration was further quantified using previously published methods ^21^. Briefly, after 36 hours of incubation, the culture supernatant was transferred to a sterile tube and centrifuged at a rate of 10,000 g for 30 minutes. The supernatant was filtered using a 0.45 µm Millipore filter and transferred to 96 new well microplates. The absorbance value of the supernatant containing pyocyanin was measured at a wavelength of 690 nm, then the pyocyanin concentration was calculated using the following equation ^21^.

Concentration of pyocyanin = A _690_ nm (A _690_ nm of sample - A _690_ nm of blank) / ε * d

ε = extinction coefficient (pyocyanin at A _690_ nm = 4310 M ^-1^cm ^-1^)

d = path length (0.23 cm for 96 well microplate)

### Statistical analysis

The data in this study were presented as the percentage of bacterial viability and pyocyanin concentration in the *P. aeruginosa* culture supernatant. All data were tested for normality using the Shapiro-Wilk and the Levene Test for homogeneity using SPSS Statistic v20. Furthermore, one-way ANOVA and Games-Howell parametric analysis were performed for bacterial cell viability data; and parametric one-way ANOVA followed by Tukey HSD on pyocyanin concentration data.

## Results

### Antibacterial activity of royal jelly against *P. aeruginosa*

**Figure 1.**
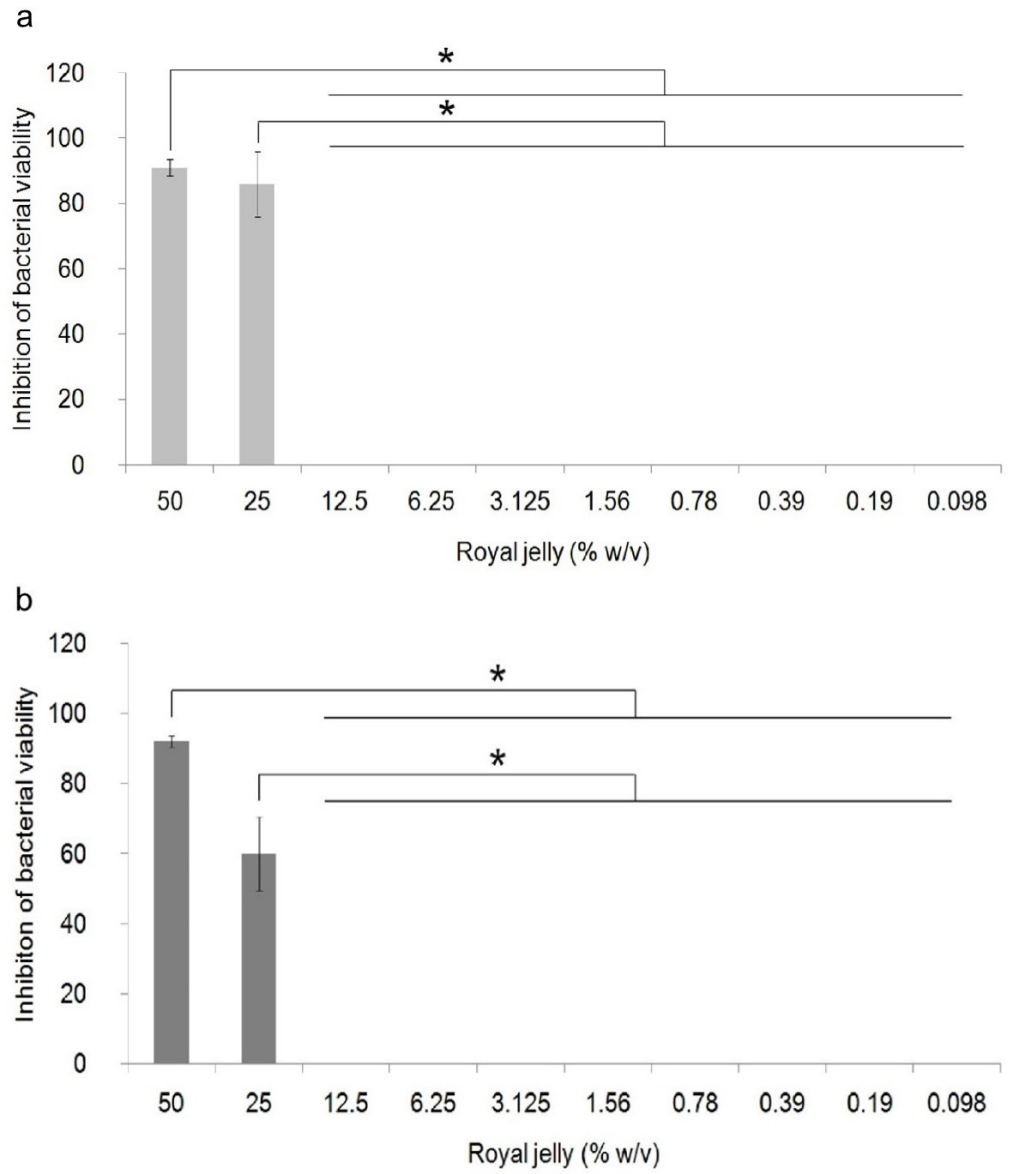
Percentage of inhibition of growth of *P. aeruginosa* bacteria (1.5 × 10 ^5^ CFU/well). Bacterial cultures were incubated with varying concentrations of royal jelly for 18 hours at 37°C. Royal jelly 50% and 25% inhibit bacterial growth. The difference is based on the results of the Games-Howell analysis at the significance value (*) *p* <0.05. ATCC ^®^ 10145™ ( **a**) strain; clinical isolate ( **b**).

The antibacterial activity of royal jelly against the two strains of *P. aeruginosa* is shown in Figure 1. Data on the percentage of bacterial growth inhibition shows normal distribution data ( *p*>0.05), but has a non-homogeneous variant ( *p*<0.05). One-way ANOVA showed a significant difference in the percentage of growth inhibition in *P. aeruginosa* ATCC ^®^ 10145™ ( *p* = 0.000) and *P. aeruginosa* clinical isolate ( *p* = 0.000) between royal jelly treatment groups and negative control. In this study, it was proven that royal jelly can inhibit the viability of both *P. aeruginosa* strains starting from a concentration of 25%. The results of the multi-comparison analysis showed that there was no significant difference between the concentrations of 25% and 50% and significant differences were identified between the concentrations of 25% and 50% with 12.5% to 0.098% in both strains. It can be concluded that the MIC for both strains is 25%.

### Exposure to subinhibitory royal jelly concentrations induced increased pyocyanin production in *P. aeruginosa*

**Figure 2.**
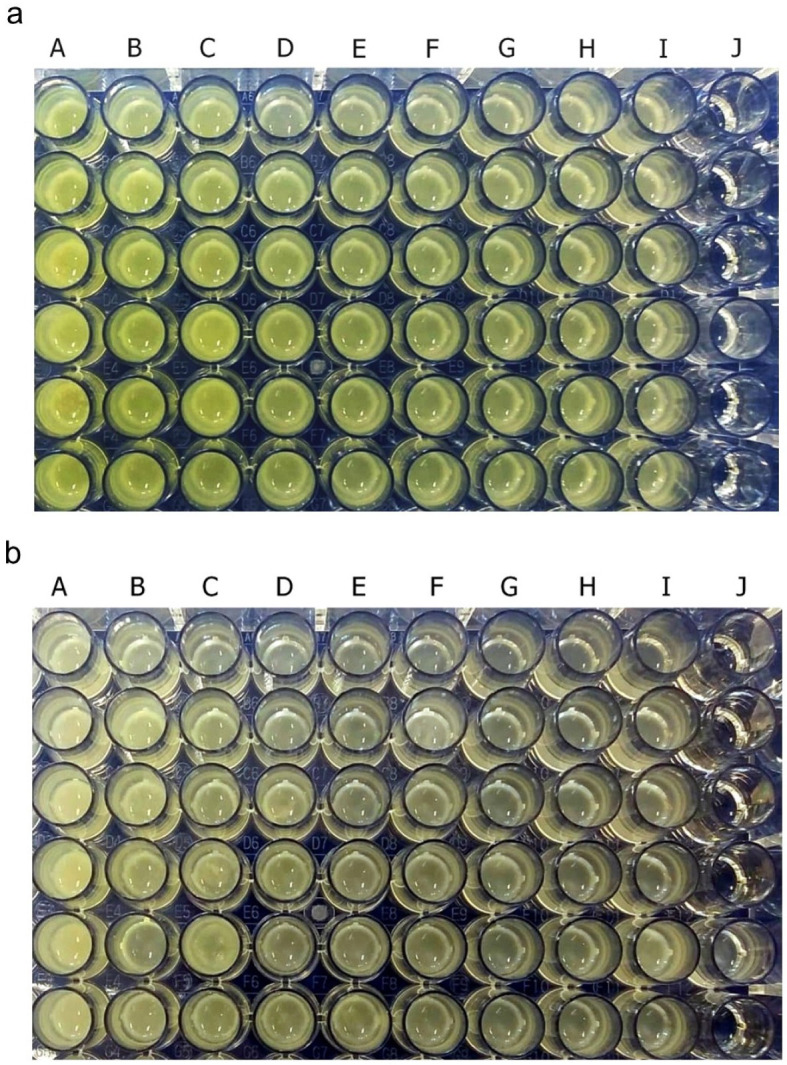
Pyocyanin was identified from the green color of the *P. aeruginosa* culture supernatant after 36 h incubation at 37°C. *P. aeruginosa* bacteria (1.5 × 10 ^5^ CFU/well) ATCC ^®^ 10145™ ( **a**); clinical isolate ( **b**). Royal jelly 12.5% (A); 6.25% (B); 3,125% (C); 1.56% (D); 0.78% (E); 0.39% (F); 0.19% (G); 0.098% (H); 0% (I); No treatment (J).

Pyocyanin was identified as green in culture supernatant *P. aeruginosa* ATCC ^®^10145™ and clinical isolate. After 36 hours of incubation, pyocyanin production was increased in the stimulated culture group with subinhibitory concentrations below 25%. The intensity of green color in the culture medium increased with the increase in the concentration of royal jelly ( Figure 2). The change in the color intensity of the culture supernatant was consistent with the results of the pyocyanin concentration measurement.

**Figure 3.**
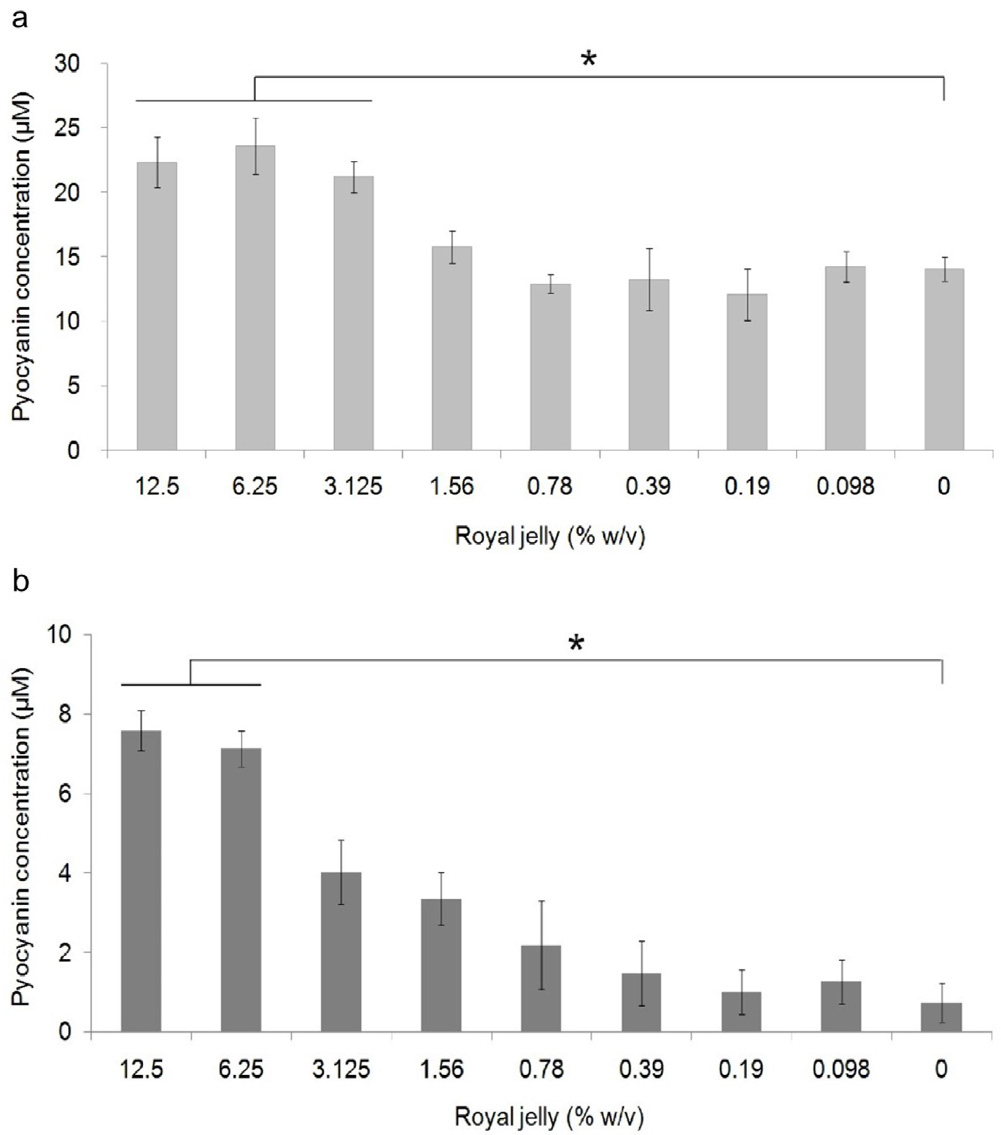
Pyocyanin production in *P. aeruginosa* bacteria (1.5 × 10 ^5^ CFU/well) after 36 hours of exposure to various concentrations of royal jelly. Pyocyanin production increased due to exposure to sub-inhibitory royal jelly concentrations of 6.25% and decreased at lower royal jelly concentrations. The difference was based on the results of the Tukey-HSD analysis at the significance value (*) *p* <0.05. ATCC ^®^ 10145™ ( **a**) strain; ( **b**) clinical isolate.

Pyocyanin concentration data in each royal jelly treatment group and negative control were the results of experiments on triplicate cultures. Figure 3 shows the average pyocyanin concentration for each group. The highest average pyocyanin concentration was identified in *P. aeruginosa* ATCC ^®^ 10145™ induced by royal jelly with a concentration of 6.25%, which was 23.59 μM, while the lowest mean was identified in clinical isolates of *P. aeruginosa* without exposure to royal jelly, which was 0.7 μM. The pyocyanin concentration of *P. aeruginosa* ATCC ^®^ 10145™ was seen to be higher than clinical isolate in the same concentration in all treatment groups.

Pyocyanin concentration data both on ATCC ^®^ 10145™ and clinical isolate in all groups were normally distributed ( *p*>0.05) and homogeneous ( *p*>0.05). There was a significant difference in the concentration of pyocyanin ATCC ^®^ 10145™ ( *p* = 0.000) and clinical isolate ( *p* = 0.000) between the treatment groups. The results of multiple comparison analysis of Tukey-HSD on *P. aeruginosa* cultures of ATCC ^®^ 10145™ showed a significant difference between the royal jelly groups with concentrations of 0% with 12.5%, 6.25%, and 3.125%. In addition, a significant difference in pyocyanin concentrations in clinical isolate was found between the 0% royal jelly group with 12.5% and 6.25%. 

## Discussion

The antibacterial effect of royal jelly has been widely reported by previous researchers ^15– 17^. The ability of royal jelly to inhibit the growth of *P. aeruginosa* is thought to be related to the variety and concentration of its antibacterial protein. Royal jelly components that have been identified as having antibacterial activity are major royal jelly protein-1 (MRJP-1), Jelleine I–III, royalicin, and 10-hydroxy-2-decenoic (10-HDA) ^15, 17, 22^.

This study showed that royal jelly concentrations of 25% and 50% had antibacterial activity against *P. aeruginosa* ATCC ^®^ 10145™ and clinical isolate. The results of this observation are different from previous studies that showed *P. aeruginosa* growth could be inhibited at concentrations >50% ^18^. This difference is thought to be closely related to differences in geographical location, botanical origin, climate, and storage conditions of royal jelly, which affect the antibacterial component of royal jelly ^23^. Previous studies have shown that royal jelly originating from different geographic and botanical locations affects the quantity of 10-HDA. Royal jelly originating from tropical climates is reported to contain lower concentrations of 10-HDA than cold climates ^23^. The higher temperature and longer storage time also resulted in a significant reduction in the quantity of MRJP1 ^24^. However, the bacterial strains studied probably also had an effect, as previously reported there was a variable response between clinical isolates and standard bacteria ^18, 19^.

*Pyocyanin* is an indicator of the pathogenicity of *P. aeruginosa*. To our knowledge, this study report is the first to demonstrate a dualism effect of royal jelly on *P. aeruginosa*. The subinhibitory concentration of royal jelly amplify the effect of an autoinducer. It was able to increase the production of pyocyanin in ATCC ^®^ 10145™ and clinical isolates to protect and maintain their survival ^13^. The pyocyanin concentration in the ATCC ^®^ 10145™ strain appeared to be significantly higher than the clinical isolates. This observation is in accordance with previous studies that found the ATCC ^®^ 10145™ strain produced more pyocyanin than the clinical isolate strains from active ulcerative keratitis patients ^25^. The presence of *phzM* and *phzS* genes was thought to affect the concentration of pyocyanin produced ^26^. This was proven by previous studies that the *phzM* and *phzS* gene expression of multidrug resistance (MDR) clinical isolate *P. aeruginosa* was lower leading to less pyocyanin production than non-MDR isolates and PAO1 strains ^27^. Some clinical isolates were also reported not to have the genes so that these bacteria cannot produce pyocyanin ^26^. Other research results also showed that the pyocyanin concentration of ATCC ^®^ 10145™ strains is higher than that of PAO1 and PA14 strains after incubation for 60 hours ^28^. It is estimated that ATCC ^®^ 10145™ is one of the strong pyocyanin producing strains. However, other virulence factors possessed by this strain were lower than the clinical isolate strains so that they were considered less virulent ^25^.

Various virulence factors, including pyocyanin are generally associated with the quorum sensing mechanism ^29^. Quorum sensing refers to the communication process between microbial cells using autoinducer molecules ^30^. One of the autoinducer molecules that plays an important role in the regulation of pyocyanin production is the pseudomonas quinolone signal (PQS). Mutation of the PQS gene results in reduced pyocyanin production ^31^. When bacterial cells are exposed to exogenic stress, such as an antibacterial agent that can threaten their survival, the bacteria immediately respond to the stimulus by inducing the production of PQS which is responsible for activating various genes involved in the production of virulence factors, including pyocyanin ^29, 32^. Although the effect of royal jelly subinhibitor concentration on this autoinducer molecule is not yet known, several studies have reported that the increase in pyocyanin production is closely related to the effect of subinhibitor antibiotics that increase PQS gene expression ^33^. It is thought that this is the cause of increased pyocyanin production at subinhibitory concentrations.

The increase in pyocyanin production in *P. aeruginosa* bacteria will have implications for the mechanism of bacterial attachment and biofilm formation. Apart from its production, which is closely related to the quorum sensing mechanism, pyocyanin is also a signaling factor in the quorum sensing process itself. This was identified from the results of research on *P. aeruginosa* PAO1 and PA14 ^34^. In addition, the increase in pyocyanin is likely to have an impact on the activity of bacteria to produce extracellular DNA (eDNA). Extracellular DNA is an important part of extracellular polymeric substance (EPS) which is the main component of the biofilm matrix. The increase in EPS production is very beneficial for the bacterial attachment process and subsequently the formation of biofilms. Pyocyanin can induce eDNA production in low level pyocyanin-producer strains, PAO1 and pyocyanin-deficient strains, PA14. In this study, it was proven that pyocyanin caused an increase in the production and release of eDNA, which is the main component in forming and stabilizing bacterial biofilms ^35^.

The increase in pyocyanin production induced by subinhibitory royal jelly concentrations in *P. aeruginosa* ATCC ^®^ 10145™ and clinical isolates in this study is an interesting phenomenon. Although, subinhibitory royal jelly concentrations were not effective in inhibiting the growth of these bacteria, on the other hand, they increased production of pyocyanin virulence factors. This has inspired the alleged biphasic nature of royal jelly which has antibacterial potential, but at different exposure concentrations, it can induce the production of *P. aeruginosa* bacteria virulence factors. This phenomenon leads us to think that researchers, as well as medical practitioners, should be careful in determining the concentration of royal jelly for its antibacterial research purposes or its therapeutic potential. This of course requires further research on the mechanisms associated with bacterial response to subinhibitory concentrations of royal jelly.

## Conclusions

Royal jelly at a concentration of 25% was only able to inhibit the growth of *P. aeruginosa* bacteria, but at subinhibitory concentrations it could increase *pyocyanin* production in *P. aeruginosa* strain ATCC ^®^ 10145™ and clinical isolate. Based on the results of this study, we suggest selecting the appropriate dose or concentration for the purpose of inhibiting the growth and production of *P. aeruginosa* virulence factors.

## Data availability

### Underlying data

Figshare: Pseudomonas aeruginosa pyocyanin, https://doi.org/10.6084/m9.figshare.13247429.v1 ^36^.

Data are available under the terms of the Creative Commons Attribution 4.0 International license (CC-BY 4.0).
