# Enhancement of pyocyanin production by subinhibitory concentration of royal jelly in *Pseudomonas aeruginosa*

**DOI:** 10.12688/f1000research.27915.4

**Published:** 2021-12-07

**Authors:** Dina Auliya Amly, Puspita Hajardhini, Alma Linggar Jonarta, Heribertus Dedy Kusuma Yulianto, Heni Susilowati

**Affiliations:** 1 Master of Dental Sciences Program, Faculty of Dentistry, Universitas Gadjah Mada, Sleman, Yogyakarta, 55281, Indonesia; 2 Department of Oral Biology, Faculty of Dentistry, Universitas Gadjah Mada, Sleman, Yogyakarta, 55281, Indonesia; 3 Department of Dental Biomedical Sciences, Faculty of Dentistry, Universitas Gadjah Mada, Sleman, Yogyakarta, 55281, Indonesia

**Keywords:** royal jelly, antibacterial effect, Pseudomonas aeruginosa, pyocyanin

## Abstract

**Background:** *Pseudomonas aeruginosa*, a multidrug-resistant Gram-negative bacterium, produces pyocyanin, a virulence factor associated with antibiotic tolerance. High concentrations of royal jelly have an antibacterial effect, which may potentially overcome antibacterial resistance. However, in some cases, antibiotic tolerance can occur due to prolonged stress of low-dose antibacterial agents. This study aimed to investigate the effect of subinhibitory concentrations of royal jelly on bacterial growth, pyocyanin production, and biofilm formation of *P. aeruginosa*.

**Methods:** *Pseudomonas aeruginosa *ATCC 10145 and clinical isolates were cultured in a royal jelly-containing medium to test the antibacterial activity. Pyocyanin production was observed by measuring the absorbance at 690 nm after 36 h culture and determined using extinction coefficient 4310 M-1 cm-1. Static microtiter plate biofilm assay performed to detect the biofilm formation, followed by scanning electron microscopy.

**Results: **Royal jelly effectively inhibited the viability of both strains from a concentration of 25%. The highest production of pyocyanin was observed in the subinhibitory concentration group 6.25%, which gradually decreased along with the decrease of royal jelly concentration. Results of one-way ANOVA tests differed significantly in pyocyanin production of the two strains between the royal jelly groups. Tukey HSD test showed concentrations of 12.5%, 6.25%, and 3.125% significantly increased pyocyanin production of ATCC 10145, and the concentrations of 12.5% and 6.25% significantly increased production of the clinical isolates. Concentrations of 12.5% and 6.125% significantly induced biofilm formation of *P. aeruginosa* ATCC 10145, in line with the results of the SEM analysis.

**Conclusions: **The royal jelly concentration of 25% or higher inhibits bacterial growth; however, the subinhibitory concentration increases pyocyanin production and biofilm formation in *P. aeruginosa* **.** It is advisable to determine the appropriate concentration of royal jelly to obtain beneficial virulence inhibiting activity.

## Introduction

*Pseudomonas aeruginosa* ( *P. aeruginosa*) is transitional pathogen. This Gram-negative bacillus can be involved in seriously opportunistic-systemic-infections, mainly in immunocompromised patients ^1– 3^. These bacteria are often found in the dental unit waterlines which allows the transmission of these bacteria into the oral cavity ^4^. As an opportunist pathogen, *P. aeruginosa* is also frequently involved in oral infections, such as necrotizing ulcerative gingivitis, periodontitis, and mandibular osteomyelitis ^5– 7^. Although the mechanism is not clear yet, its presence in the oral cavity has been shown to result in systemic infections, such as nosocomial pneumonia ^8^.

Based on the reports from several clinical cases, the infection caused by *P. aeruginosa* bacteria can be fatal. Treatment of *P. aeruginosa* infection is sometimes ineffective, which is closely related to the number of virulence factors possessed by the bacteria ^9^. The bacterial cell surface components and some secretory products are important virulence factors of *P. aeruginosa*, one of which is pyocyanin ^10^. Pyocyanin is a cytotoxic pigment from the Phenazine group of compounds that can facilitate biofilm development, cause pro-inflammatory effects, and result in host cell death ^11^.

The resistance of *P. aeruginosa* to various spectrums of antibiotics creates difficulties in handling the infection it causes ^12^. It has been reported recently that the administration of antibiotics below the minimum inhibitory concentration (MIC) can cause specific bacterial responses, such as an increase in pyocyanin production in *P. aeruginosa*. PAO1 and P14 are the attempts by the bacteria to survive under antibiotic stress ^13^. This certainly motivates researchers to further analyse the infection they cause, and find the appropriate antibiotic concentration or dose to overcome the problem.

Royal jelly is a natural bee product produced by hypopharyngeal and mandibular glands of worker bees. This substance is a "superfood" for the queen bee. It is widely used as a nutrient-rich supplement to overcome various health conditions such as inflammation, bacterial infection, tumor, allergy, and antioxidant and immunomodulatory effects ^14, 15^. Royal jelly proteins, such as Jelleine, major royal jelly protein-1 (MRJP1), and royalicin are known to have antibacterial effects against *P. aeruginosa*. Major royal jelly protein-1 and Jelleine can interfere with the permeability of the outer membrane of the cell, causing the loss of vital contents of bacterial cells, which in turn causes cell death. Cationic antimicrobial peptides, such as royalicin, are known to also interfere with cell membrane permeability in various Gram-positive and Gram-negative bacteria, such as *P. aeruginosa* ^16– 18^. Results of previous studies have shown that royal jelly can inhibit the growth of *P. aeruginosa*. In this study, royal jelly showed inhibition of the growth of *P. aeruginosa* ATCC 27853 ^19^. In addition, it has also been known that royal jelly in various concentrations can inhibit the nonspecific attachment of *P. aeruginosa* ATCC 27853 ^20^, but so far, the effect of the subinhibitory concentration of royal jelly against these bacteria is unknown. Furthermore, as pyocyanin is an indicator of the pathogenicity of *P. aeruginosa* strains, the aim of this study was to determine the effect of subinhibitory royal jelly concentration on pyocyanin production and biofilm formation in representative strains of a high level pyocyanin-producer *P. aeruginosa* ATCC 10145 and clinical isolate.

## Methods

This *in vitro* laboratory experimental research was done at the Integrated Research Laboratory of the Faculty of Dentistry, Universitas Gadjah Mada, Yogyakarta. All research procedures have been approved by the Ethics Committee of the Faculty of Dentistry, Universitas Gadjah Mada, Yogyakarta (No. 00393/KKEP/ FKG-UGM/EC/2020).

Royal jelly used in this study was obtained from Nusukan, Surakarta, Central Java, Indonesia and harvested in May–October 2019. Royal jelly produced by *Apis mellifera* bees live in kapok tree ( *Ceiba pentandra*) and sono wood ( *Dalbergia latifolia*). *Apis mellifera* bee species have been identified through previous research ^20^. Royal jelly 5.5 grams was dissolved in 10 ml of cold phosphate buffered saline (PBS), then homogenized using a magnetic stirrer (24 hours, 4°C). The royal jelly solution was centrifuged (12,000 g, 45 minutes, 4°C), then the supernatant was taken and checked the pH. The results of pH measurements with a pH meter showed a pH of 3.79. Furthermore, the supernatant was filtered using 0.45 µm Millipore to produce 55% royal jelly and stored at a temperature of 4–8°C ^21^.

*Pseudomonas aeruginosa* ATCC 10145 (Thermo Scientific) was obtained from the Integrated Research Laboratory of the Faculty of Dentistry, Universitas Gadjah Mada. A clinical isolate of *P. aeruginosa* derived from patient sputum was obtained from the Laboratory of Microbiology, Faculty of Medicine, Public Health and Nursing, Universitas Gadjah Mada. Identification of the clinical isolate was carried out using API 2ONE (bioMérieux, Inc). Both of these strains were each inoculated in Luria Bertani broth (LB; Himedia Laboratories) and incubated at 37°C for 24 hours. After that, the culture was centrifuged at 3000 rpm for 15 minutes and then resuspended using 0.98% NaCl to obtain a bacterial concentration equivalent to 1.5 × 10 ^5^ CFU/ml.

### Measurement of the effect of royal jelly on the viability of *P. aeruginosa*

A sterile 55% w/v royal jelly solution was diluted in brain heart infusion (BHI; Himedia Laboratories) broth to obtain a concentration of 50% and then serial dilution was performed in 96 well microplates. A total of 5 µl of the *P. aeruginosa* ATCC 10145 suspension or clinical isolate bacteria (1.5 × 10 ^5^ CFU/ml) was inoculated in all groups, except the groups that had been determined as blanks. The culture was then incubated at 37°C for 18 hours. After that, the microplate was scanned using the Spark® Multimode Microplate Reader (Tecan trading AG) to measure optical density (OD) using a 600 nm wavelength. The percentage of bacterial viability inhibition was determined based on the OD value of the treatment group against the control. The bacterial viability assay experiments were carried out in quadruplicate.

### Analysis of the effect of royal jelly on pyocyanin production

Royal jelly solution was diluted into sterile BHI broth to get the concentration of 12.5%, 6.25%, 3.125%, 1.56%, 0.78%, 0.39%, 0.19%, and 0.098% w/v. Both strains of *P. aeruginosa* were cultured on BHI broth containing various concentrations of royal jelly as treatment and BHI broth only as a blank. The cultures were incubated at 37°C for 36 hours, then the pyocyanin production of each strain was observed visually, which appeared green in the culture supernatant. The pyocyanin concentration was further quantified using previously published methods ^22^. Briefly, after 36 hours of incubation, the culture supernatant was transferred to a sterile tube and centrifuged at a rate of 10,000 g for 30 minutes. The supernatant was filtered using a 0.45 µm Millipore filter and transferred to fresh 96-well microplates. The absorbance value of the supernatant containing pyocyanin was measured at a wavelength of 690 nm, then the pyocyanin concentration was calculated using the following equation ^22^. The pyocyanin experiments were performed in triplicate.

Concentration of pyocyanin = A _690_ nm (A _690_ nm of sample - A _690_ nm of blank) / ε * d

ε = extinction coefficient (pyocyanin at A _690_ nm = 4310 M ^-1^cm ^-1^)

d = path length (0.23 cm for 96 well microplate)

### Analysis of the effect of royal jelly on biofilm formation

Static microtiter plate biofilm assay with crystal violet staining was performed to detect the association between pyocyanin production and biofilm formation. We have used *P. aeruginosa* ATCC 10145 as this strain is more responsive in producing pyocyanin when compared to the clinical isolate strains we previously used. Experiments were carried out on a 96-well U bottom microtiter plate (Iwaki, Japan). *P. aeruginosa* ATCC 10145 (1.5 × 10 ^8^ CFU/ml) inoculated in 100 µl of LB broth as negative control and on culture media containing royal jelly with final concentration at 25%, 12.5%, 6.25%. The cultures were incubated aerobically at 37°C for 24 hours. After completing the incubation period, the culture rinsed three times using 200 µL of 1x PBS, followed by staining using 200 µL of 0.1% crystal violet for 15 minutes at room temperature. Following incubation, 1x PBS was used to rinse the biofilm, followed by exposure of the biofilm mass with 200 µl of absolute methanol for 15 minutes at ambient temperature. Afterward, 100 µl of the biofilm mass solution was transferred to a flat bottom 96-well microplate and the absorbance was read using a microplate reader at a wavelength of 540 nm (Multiskan Sky, Thermoscientific). The percentage change in biofilm mass of each treatment group was calculated from the absorbance value using the formula we previously used ^21^. The experiments were carried out in triplicate.

### Analysis of biofilm architecture 

Scanning electron microscopy was performed to analyse the architecture of the biofilm mass of *P. aeruginosa* ATCC 10145. The bacterial suspension (1.5 × 10 ^8^ CFU/ml) was inoculated into a sterile tube containing LB broth culture media mixed with royal jelly with a final concentration of 25%, 12.5%, 6.25%. Chlorhexidine gluconate 0.2% was used as a positive control. Meanwhile, for the negative control group, the bacteria were inoculated into LB broth only. Five sterile composite resin discs with a 5 mm diameter and 2 mm height were prepared by using an aseptic method as a medium for adhering to bacteria. The discs were immersed in each tube containing culture media mixed with royal jelly and incubated aerobically at 37°C for 24 hours to provide an opportunity for bacterial biofilm growth. On the following day, each resin disc was taken and processed for qualitative analysis using SEM (JSM – 6510LA, JEOL Ltd, Japan) to investigate the biofilm-mass development on the surface of discs for respective royal jelly concentration.

### Statistical analysis

The data in this study were presented as the percentage of bacterial viability, biofilm mass, and pyocyanin concentration in the P. aeruginosa culture supernatant. Data were analysed by using the Shapiro-Wilk for normality, followed by Levene's Test for homogeneity using SPSS Statistic v20. Furthermore, one-way ANOVA and Games-Howell parametric analysis were performed for bacterial cell viability and biofilm mass formation data; and parametric one-way ANOVA followed by Tukey HSD on pyocyanin concentration data.

## Results

### Antibacterial activity of royal jelly against *P. aeruginosa*

**Figure 1.**
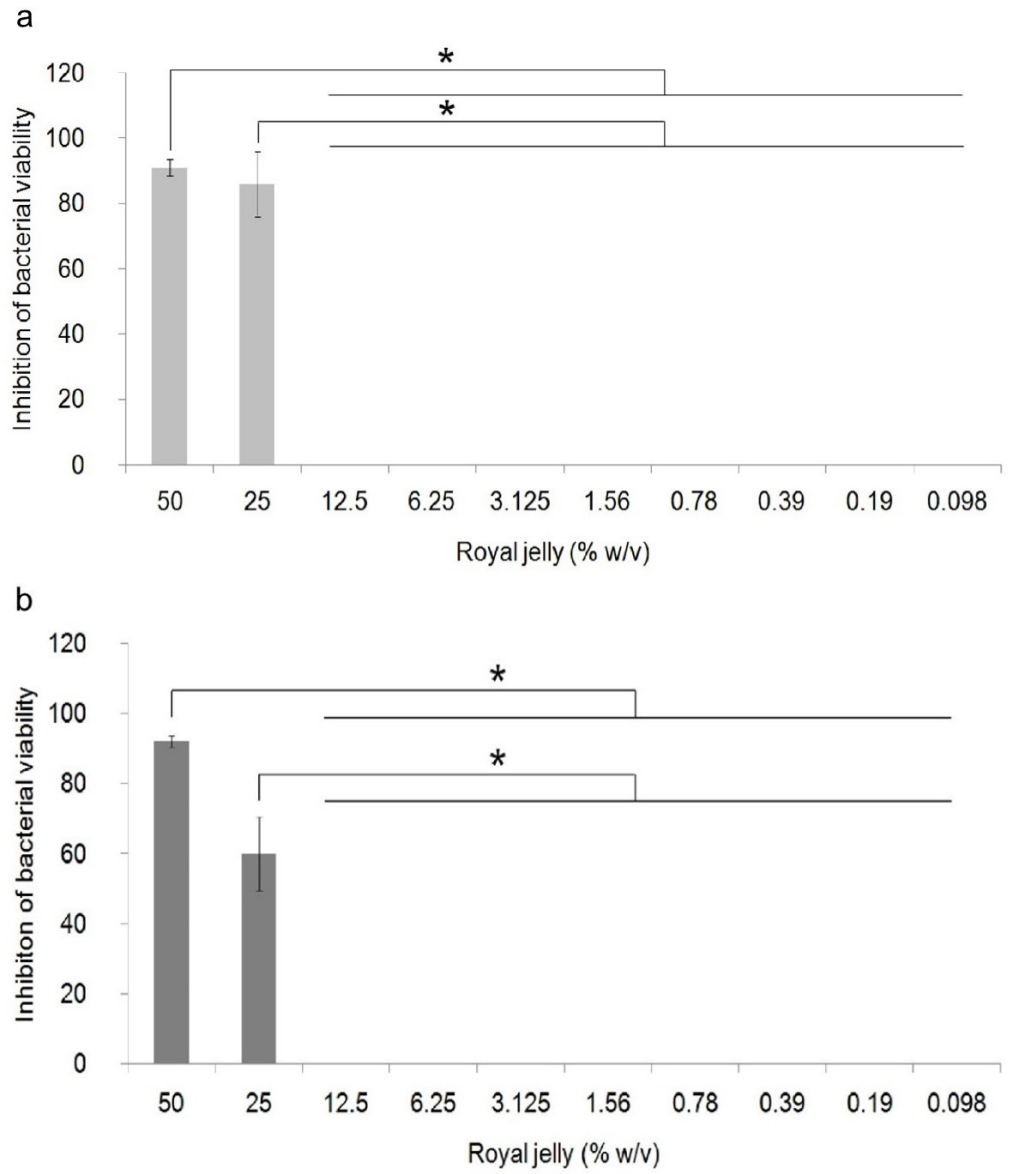
Percentage of inhibition of growth of *P. aeruginosa* bacteria (1.5 × 10 ^5^ CFU/well). Bacterial cultures were incubated with varying concentrations of royal jelly for 18 hours at 37°C. Royal jelly 50% and 25% inhibit bacterial growth. The difference is based on the results of the Games-Howell analysis at the significance value (*) *p* <0.05. ATCC 10145 ( **a**) strain; clinical isolate ( **b**).

The antibacterial activity of royal jelly against the two strains of *P. aeruginosa* is shown in Figure 1. Data on the percentage of bacterial growth inhibition shows normal distribution data ( *p*>0.05), but has a non-homogeneous variant ( *p*<0.05). One-way ANOVA showed a significant difference in the percentage of growth inhibition in *P. aeruginosa* ATCC ^®^ 10145™ ( *p* = 0.000) and *P. aeruginosa* clinical isolate ( *p* = 0.000) between royal jelly treatment groups and negative control. In this study, it was proven that royal jelly can inhibit the viability of both *P. aeruginosa* strains starting from a concentration of 25%. The results of the multi-comparison analysis showed that there was no significant difference between the concentrations of 25% and 50% and significant differences were identified between the concentrations of 25% and 50% with 12.5% to 0.098% in both strains. In clinical isolate, 25% royal jelly caused 60% death in the bacterial population; meanwhile, in *P. aeruginosa* ATCC 10145 this concentration caused growth inhibition of 85%. We therefore categorized royal jelly concentrations below 25% as subinhibitory concentrations.

### Exposure to subinhibitory royal jelly concentrations induced increased pyocyanin production in *P. aeruginosa*

**Figure 2.**
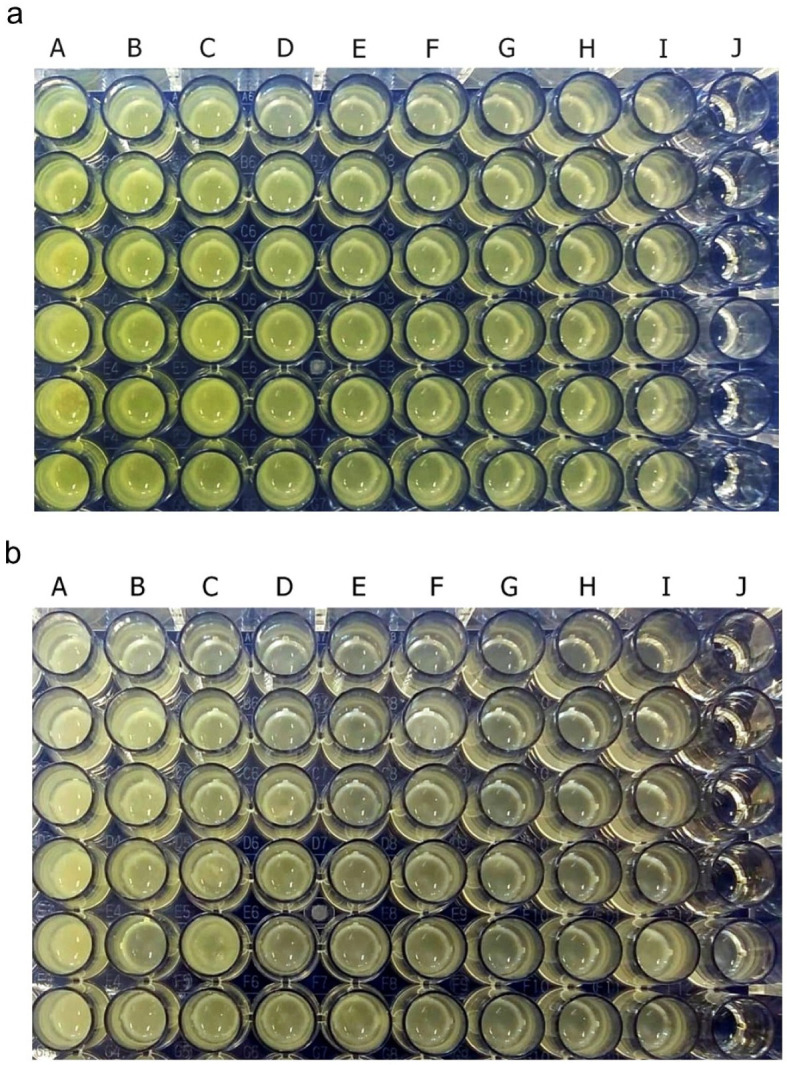
Pyocyanin was identified from the greenish colour of the *P. aeruginosa* culture supernatant after 36 h incubation at 37°C. *P. aeruginosa* bacteria (1.5 × 10 ^5^ CFU/well) ATCC 10145 ( **a**); clinical isolate ( **b**). Royal jelly 12.5% (A); 6.25% (B); 3,125% (C); 1.56% (D); 0.78% (E); 0.39% (F); 0.19% (G); 0.098% (H); 0% (I); No treatment (J).

Pyocyanin was identified as greenish colour in culture supernatant *P. aeruginosa* ATCC 10145 and clinical isolate. After 36 hours of incubation, pyocyanin production was increased in the stimulated culture group with subinhibitory concentrations below 25%. The intensity of green colour in the culture medium increased with the increase in the concentration of royal jelly ( Figure 2). The change in the colour intensity of the culture supernatant was consistent with the results of the pyocyanin concentration measurement.

**Figure 3.**
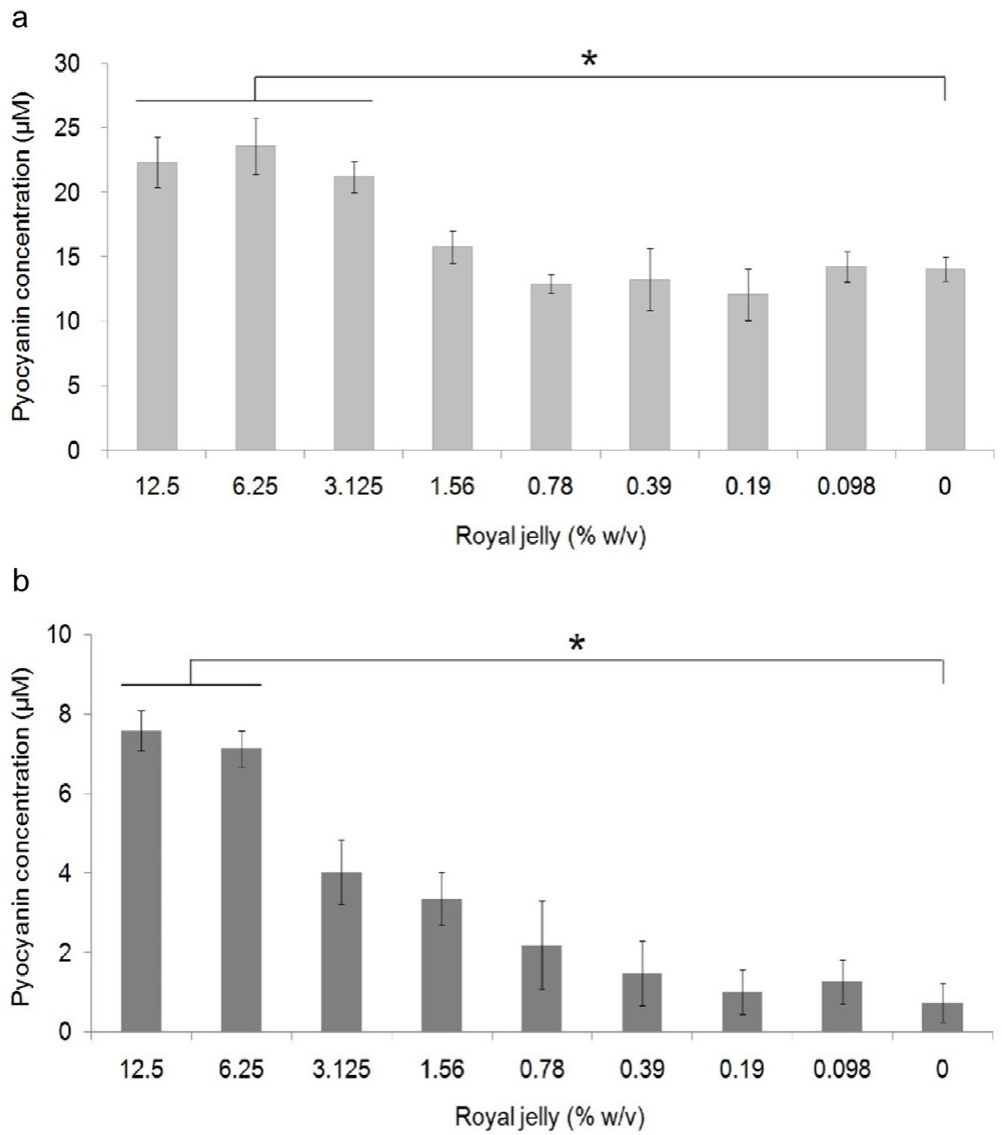
Pyocyanin production in *P. aeruginosa* bacteria (1.5 × 10 ^5^ CFU/well) after 36 hours of exposure to various concentrations of royal jelly. Pyocyanin production increased due to exposure to sub-inhibitory royal jelly concentrations of 6.25% and decreased at lower royal jelly concentrations. The difference was based on the results of the Tukey-HSD analysis at the significance value (*) *p* <0.05. ATCC 10145 ( **a**) strain; ( **b**) clinical isolate.

Pyocyanin concentration data in each royal jelly treatment group and negative control were the results of experiments on triplicate cultures. Figure 3 shows the average pyocyanin concentration for each group. The highest average pyocyanin concentration was identified in *P. aeruginosa* ATCC 10145 induced by royal jelly with a concentration of 6.25%, which was 23.59 μM, while the lowest mean was identified in clinical isolate of *P. aeruginosa* without exposure to royal jelly, which was 0.7 μM. The pyocyanin concentration of *P. aeruginosa* ATCC 10145 was seen to be higher than clinical isolate in the same concentration in all treatment groups.

Pyocyanin concentration data both on ATCC 10145 and clinical isolate in all groups were normally distributed ( *p* > 0.05) and homogeneous ( *p* > 0.05). There was a significant difference in the concentration of pyocyanin ATCC 10145 ( *p* = 0.000) and clinical isolate ( *p* = 0.000) between the treatment groups. The results of multiple comparison analysis of Tukey-HSD on *P. aeruginosa* cultures of ATCC 10145 showed a significant difference between the royal jelly groups with concentrations of 0% with 12.5%, 6.25%, and 3.125%. In addition, a significant difference in pyocyanin concentrations in clinical isolate was found between the 0% royal jelly group with 12.5% and 6.25%. 

### Subinhibitory royal jelly concentrations increased biofilm formation in *P. aeruginosa*

**Figure 4.**
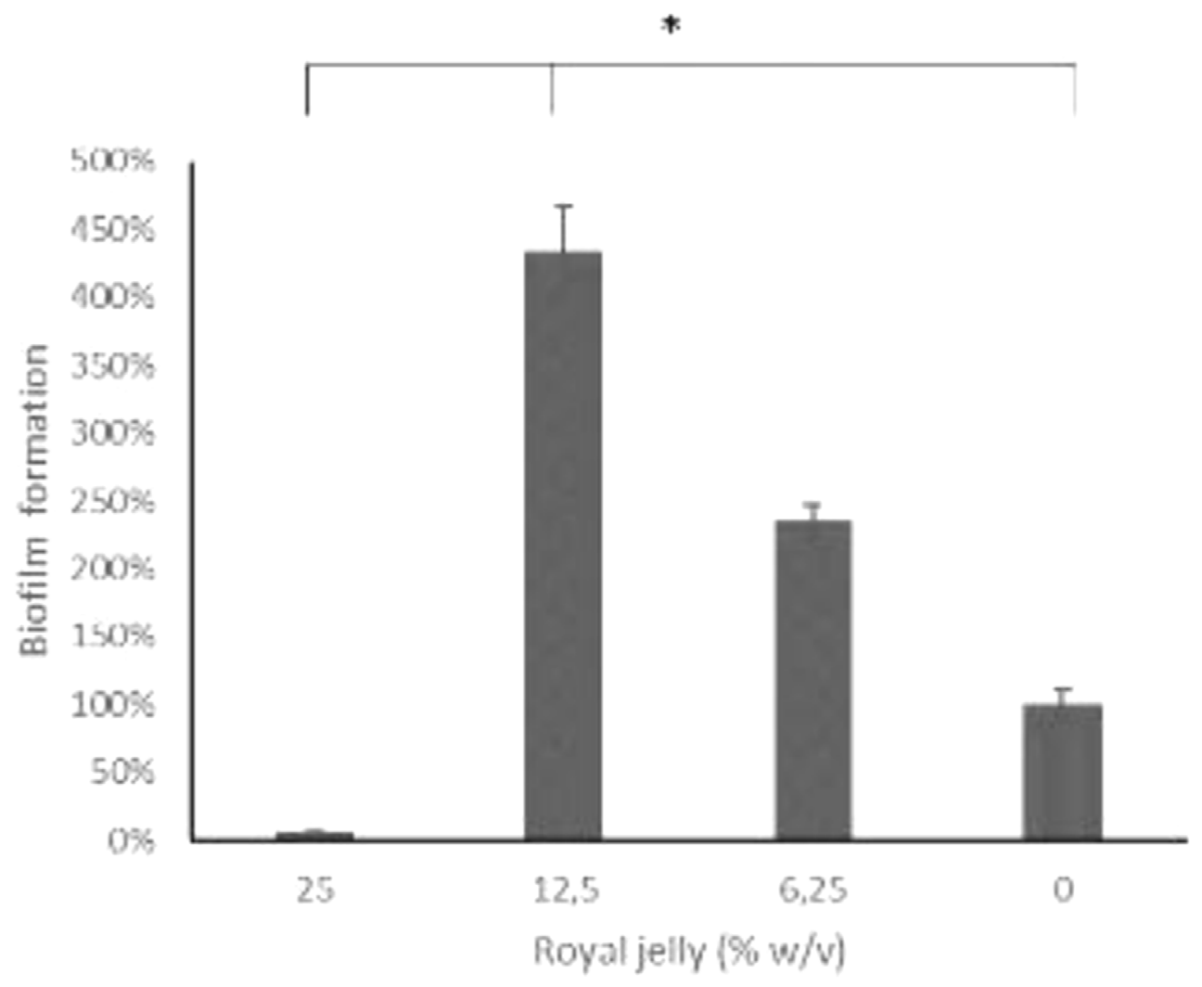
The 12.5% royal jelly increased the formation of *P. aeruginosa* biofilm stronger than 25% and 6.25% royal jelly. ATCC 10145.

The results of the biofilm mass test using a static microtiter plate biofilm assay were presented as the relative percentage value of the biofilm mass in the treatment group to the negative control ( Figure 4).

Figure 4 illustrates the peculiarity of 12.5% royal jelly that induced biofilm mass formation up to four times as much (434.06%) than bacteria not exposed to royal jelly. The lower concentration of royal jelly (6.12%) also increased biofilm formation but was lower than 12.5% royal jelly (234.35%). Meanwhile, the concentration of 25% suppressed the bacterial growth so that less biofilm compared to the other groups, around 5.85% relative to the negative control group. Welch ANOVA analysis has shown significant differences across groups ( *p* < 0.05). In addition, the results of multiple comparisons using Games-Howell showed a significant difference between royal jelly 12.5% and 25%, 6.25%, and the negative control group ( *p* < 0.05).

### The effect of royal jelly on *P. aeruginosa* biofilm architecture

**Figure 5.**
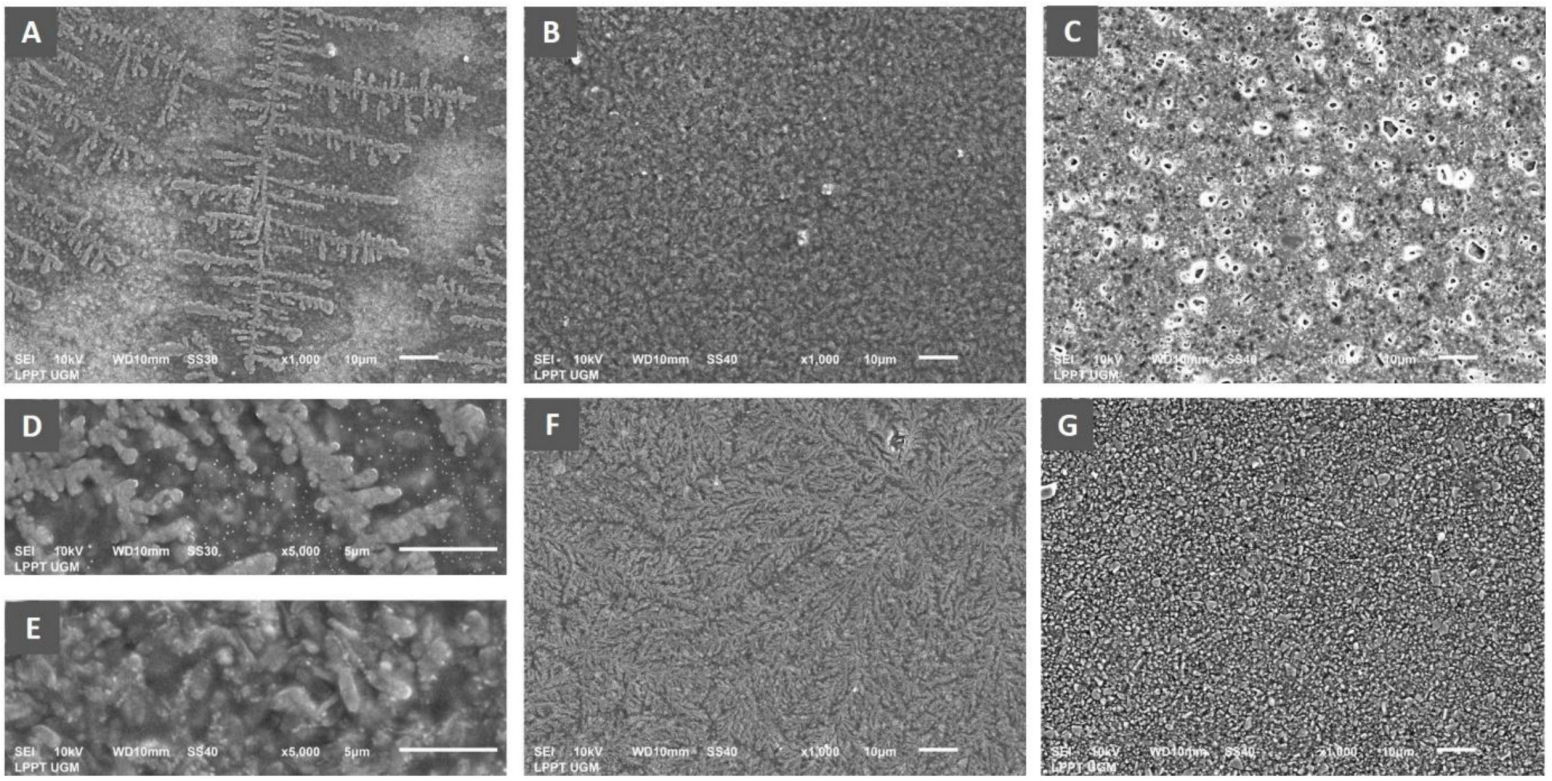
Scanning electron micrograph of *P. aeruginosa* biofilm showed the formation of specific bacterial colonies on the adhesion surface with extensions indicating bacterial motility in the group exposed to royal jelly 6.25% ( **A**), complete coverage of the adhesion surface by the bacterial biofilm mass in the treatment group with royal jelly 12.5% ( **B**). Adhesion surface was free from bacterial biofilm in the bacterial group treated with 25% royal jelly ( **C**). Figure **D** shows the biofilm architecture of the 6.25% royal jelly group, and **E** is that of the 12.5% royal jelly group at a magnification of x5000. The negative control group showed evenly distributed bacterial colonies on the adhesion surface ( **F**), while the positive control group (chlorhexidine) showed complete inhibition of biofilm formation ( **G**). Overall, the SEM results presented the highest increase in biofilm mass formation by 12.5% royal jelly. ATCC 10145 strain.

Observation of biofilm mass architecture using scanning electron microscopy has been carried out to complement the data from the measurement of biofilm quantity. The results indicated an effect of differences in royal jelly concentrations on the biofilm characteristics of *P. aeruginosa* ATCC 10145 ( Figure 5).

The x1000 magnification of the 6.25% royal jelly specimen showed a specific appearance of the fern-like bacterial colony. The x5000 magnification gives a clearer picture of the colony's shape. Meanwhile, the biofilm mass was thicker in the specimens treated with 12.5% royal jelly. Bacterial colonies covered all surfaces of the attachment media, significantly different from bacterial biofilms treated with 6.25% royal jelly. However, the biofilm mass formation inhibited in the group treated with 25% royal jelly. The subinhibitory concentrations of royal jelly increased biofilm formation; the thickest biofilm mass was in the 12.5% royal jelly treatment group.

## Discussion

The antibacterial effect of royal jelly has been widely reported by previous researchers ^16– 18^. The ability of royal jelly to inhibit the growth of *P. aeruginosa* is thought to be related to the variety and concentration of its antibacterial protein. Royal jelly components that have been identified as having antibacterial activity are major royal jelly protein-1 (MRJP-1), Jelleine I–III, royalicin, and 10-hydroxy-2-decenoic (10-HDA) ^16, 18, 23^.

This study performed the extraction method using phosphate buffer saline ^24^ that was chosen in order to dissolve MRJP1, the active components that are antibacterial or anti-adhesion. It is known that MRJP1 is a hydro soluble protein dissolved in PBS, which is higher in supernatants than in pellets ^25^. After centrifugation there were indeed pellets, and it has been found that the pellets contain more liposoluble proteins than supernatants. To ensure that the compounds contained in the PBS royal jelly extract supernatant, further research is needed. However, from the study of Furusawa *et al*., it was found that MRJP1 was contained in the supernatant of PBS royal jelly extract in quite a large amount, reaching 27.6%. The active components that are antibacterial or anti-adhesion in the supernatant of royal jelly extract in this study cannot be explained with certainty, but the royal jelly extract used in this study was thought to mainly contain MRJP1 ^26^.

This study showed that royal jelly concentrations of 25% and 50% had antibacterial activity against *P. aeruginosa* ATCC 10145 and clinical isolate. The results of this observation are different from previous studies that showed *P. aeruginosa* growth could be inhibited at concentrations >50% ^19^. This difference is thought to be closely related to differences in geographical location, botanical origin, climate, and storage conditions of royal jelly, which affect the antibacterial component of royal jelly ^27^. Previous studies have shown that royal jelly originating from different geographic and botanical locations affects the quantity of 10-HDA. Royal jelly originating from tropical climates is reported to contain lower concentrations of 10-HDA than cold climates ^27^. The higher temperature and longer storage time also resulted in a significant reduction in the quantity of MRJP1 ^28^. However, the bacterial strains studied probably also had an effect, as previously reported there was a variable response between clinical isolates and standard bacteria ^19, 20^.

*Pyocyanin* is an indicator of the pathogenicity of *P. aeruginosa*. To our knowledge, this study report is the first to demonstrate a dualism effect of royal jelly on *P. aeruginosa*. The subinhibitory concentration of royal jelly amplify the effect of an autoinducer. It was able to increase the production of pyocyanin in ATCC 10145 and clinical isolate to protect and maintain their survival ^13^. The pyocyanin concentration in the ATCC 10145 strain appeared to be significantly higher than the clinical isolate. This observation is in accordance with previous studies that found the ATCC 10145 strain produced more pyocyanin than the clinical isolate strains from active ulcerative keratitis patients ^29^. The presence of *phzM* and *phzS* genes was thought to affect the concentration of pyocyanin produced ^30^. This was proven by previous studies that the *phzM* and *phzS* gene expression of multidrug resistance (MDR) clinical isolate *P. aeruginosa* was lower leading to less pyocyanin production than non-MDR isolates and PAO1 strains ^31^. Some clinical isolates were also reported not to have the genes so that these bacteria cannot produce pyocyanin ^30^. Other research results also showed that the pyocyanin concentration of ATCC 10145 strains is higher than that of PAO1 and PA14 strains after incubation for 60 hours ^32^. It is estimated that ATCC 10145 is one of the strong pyocyanin producing strains. However, other virulence factors possessed by this strain were lower than the clinical isolate strains so that they were considered less virulent ^29^.

Various virulence factors, including pyocyanin are generally associated with the quorum sensing mechanism ^33^. Quorum sensing refers to the communication process between microbial cells using autoinducer molecules ^34^. One of the autoinducer molecules that plays an important role in the regulation of pyocyanin production is the pseudomonas quinolone signal (PQS). Mutation of the PQS gene results in reduced pyocyanin production ^35^. When bacterial cells are exposed to exogenic stress, such as an antibacterial agent that can threaten their survival, the bacteria immediately respond to the stimulus by inducing the production of PQS which is responsible for activating various genes involved in the production of virulence factors, including pyocyanin ^33, 36^. Although the effect of royal jelly subinhibitory concentration on this autoinducer molecule is not yet known, several studies have reported that the increase in pyocyanin production is closely related to the effect of subinhibitory antibiotics that increase PQS gene expression ^37^. It is thought that this is the cause of increased pyocyanin production at subinhibitory concentrations.

The increase in pyocyanin production in *P. aeruginosa* bacteria will have implications for the mechanism of bacterial attachment and biofilm formation. Apart from its production, which is closely related to the quorum sensing mechanism, pyocyanin is also a signalling factor in the quorum sensing process itself. This was identified from the results of research on *P. aeruginosa* PAO1 and PA14 ^38^. We have found that subinhibitory concentration of royal jelly increases pyocyanin production. It has also increased biofilm mass formation. It confirmed in the strain producing high levels of pyocyanin, *P. aeruginosa* ATCC 10145. In addition, the increase in pyocyanin is likely to have an impact on the activity of bacteria to produce extracellular DNA (eDNA). Extracellular DNA is an important part of extracellular polymeric substance (EPS) which is the main component of the biofilm matrix. The increase in EPS production is very beneficial for the bacterial attachment process and subsequently the formation of biofilms. Pyocyanin can induce eDNA production in low level pyocyanin-producer strains, PAO1 and pyocyanin-deficient strains, PA14. In this study, it was proven that pyocyanin caused an increase in the production and release of eDNA, which is the main component in forming and stabilizing bacterial biofilms ^39^.

The regulation of biofilm formation and pyocyanin production involves the regulatory genes of the PQS system and the Rhl system. The mechanism of the PQS system in regulating pyocyanin synthesis has been confirmed in several strains of *P. aeruginosa* bacteria but may vary depending on each strain ^40^. The mechanism of the effect of royal jelly on these systems is not yet known. In this regard, in the future, it is important to analyse the effect of royal jelly on the expression of genes related to PQS and Rhl regulation in *P. aeruginosa* bacteria.

The increase in pyocyanin production induced by subinhibitory royal jelly concentrations in *P. aeruginosa* ATCC 10145 and clinical isolate in this study is an interesting phenomenon. Although, subinhibitory royal jelly concentrations were not effective in inhibiting the growth of these bacteria, on the other hand, they increased production of pyocyanin virulence factors. The known bee products are honey, propolis, and royal jelly. All three have antibacterial and antibiofilm properties, in addition to other therapeutic potentials in the process of tissue healing, reproduction, and infectious diseases ^15^. Manuka honey has been widely studied and proven to be antibacterial and inhibit the formation of biofilms in *P. aeruginosa*; however, bacteria derived from biofilms experienced a decrease in sensitivity to manuka honey due to the presence of variants in the bacterial population ^41^ or due to subinhibitory concentrations of manuka honey ^42^. Our results indicate that the sensitivity of *P. aeruginosa* bacteria to royal jelly may also vary, depending on the concentration of royal jelly exposed to the bacteria. At sub-inhibitory concentration, we found the increase of the pyocyanin production and biofilm growth. This has inspired the alleged biphasic nature of royal jelly which has antibacterial potential, but at different exposure concentrations, it can induce the production of *P. aeruginosa* bacteria virulence factors. This phenomenon leads us to think that researchers, as well as medical practitioners, should be careful in determining the concentration of royal jelly for its antibacterial research purposes or its therapeutic potential. This of course requires further research on the mechanisms associated with bacterial response to subinhibitory concentrations of royal jelly.

## Conclusions

Royal jelly at a concentration of 25% was able to inhibit the growth of *P. aeruginosa* bacteria, but at subinhibitory concentrations, it could increase pyocyanin production in *P. aeruginosa* strain ATCC 10145 and clinical isolate. The subinhibitory concentration of royal jelly also increased biofilm formation in *P. aeruginosa* ATCC 10145. Based on the results of this study, we suggest selecting the appropriate dose or concentration for the purpose of inhibiting the growth and production of *P. aeruginosa* virulence factors.

## Data availability

### Underlying data

Figshare: *Pseudomonas aeruginosa* pyocyanin, https://doi.org/10.6084/m9.figshare.13247429.v1 ^43^.

Data are available under the terms of the Creative Commons Attribution 4.0 International license (CC-BY 4.0).
