# Supplementary material for: Enhancement of pyocyanin production by subinhibitory concentration of royal jelly in Pseudomonas aeruginosa
Source: F1000Res. 2021 Dec 7;10:14. Originally published 2021 Jan 11. [Version 4] doi: 10.12688/f1000research.27915.4 (PMC8424461; doi:10.12688/f1000research.27915.4)
Supplement: Peer review history

# Peer review: Reviewer response for version 4

**DOI:** 10.5256/f1000research.79877.r102187

**Published:** 2022-01-20

**Authors:** Philip S. Bird

**Affiliations:** 1 School of Veterinary Science, Faculty of Science, The University of Queensland, Gatton, Qld, Australia

I still have a few issues with the latest revision.

 First paragraph of Introduction:

*"Pseudomonas aeruginosa* ( *P. aeruginosa*) is **transitional** pathogen. This Gram-negative bacillus can be involved in seriously opportunistic-systemic-infections, mainly in immunocompromised patients ^1– 3^. These bacteria are often found in the dental unit waterlines which allows the transmission of these bacteria into the oral cavity ^4^. As an opportunist pathogen, *P. aeruginosa* is also **frequently involved** in oral infections, such as necrotizing ulcerative gingivitis, periodontitis, and mandibular osteomyelitis ^5– 7^. Although the mechanism is not clear yet, its presence in the oral cavity has been shown to result in systemic infections, such as nosocomial pneumonia ^8^."

 My comment was to illustrate that Pa is a transitional bacterium in the oral cavity. It is an opportunistic pathogen. It is not a frequent oral pathogen as the references refer to case studies. A better reference to cite to illustrate Pa infections is Klochgetter and Tümmler (2017 ^1^), for example.

 Methods: "Royal jelly used in this study was obtained from Nusukan, Surakarta, Central Java, Indonesia and harvested in May–October 2019. **Royal jelly produced by *Apis mellifera* bees live in kapok tree ( *Ceiba pentandra*) and sono wood** ( *Dalbergia latifolia*). ***Apis mellifera* bee species have been identified through previous research ^20^. Royal jelly 5.5 grams** was dissolved in 10 ml…"

  

1. I’m not sure what the tree species have to do with the production of RJ - maybe say that RJ was harvested from feral bee colonies found in kapok trees etc. - but usually to collect royal jelly hives as set up so that bees produce a number of queen cells are from those royal jelly is collected. Maybe the authors could look at how royal jelly is produced, cited in reference Fratini *et al.* (2016) #14.
2. I’m not sure what the sentence with cited reference #20 means.
3. Was the RJ freeze-dried or a raw fresh extracted solution?

My comments in the review:

 Just as an addendum, while this paper is mainly a technical paper I thought it would be **of interest to readers with a few sentences on bee products** as well as royal jelly. Something on how royal jelly is produced by bees. **How and what would royal jelly be used for in treatment and what infections?**

1. It is good to see that some discussion on other bee products was included in the last paragraph of the discussion. Bee products appear to be used currently for treatment of wounds. How does this research lead to royal jelly treatments of Pa infections? Are there any current papers that can be cited?

Is the work clearly and accurately presented and does it cite the current literature?

Partly

If applicable, is the statistical analysis and its interpretation appropriate?

I cannot comment. A qualified statistician is required.

Are all the source data underlying the results available to ensure full reproducibility?

Yes

Is the study design appropriate and is the work technically sound?

Yes

Are the conclusions drawn adequately supported by the results?

Yes

Are sufficient details of methods and analysis provided to allow replication by others?

Partly

Reviewer Expertise:

NA

I confirm that I have read this submission and believe that I have an appropriate level of expertise to confirm that it is of an acceptable scientific standard, however I have significant reservations, as outlined above.

## References

[1] : Recent advances in understanding Pseudomonas aeruginosa as a pathogen. *F1000Res* .2017;6: 10.12688/f1000research.10506.1 1261 10.12688/f1000research.10506.1 28794863PMC5538032

# Peer review: Reviewer response for version 3

**DOI:** 10.5256/f1000research.77925.r92435

**Published:** 2021-10-18

**Authors:** Philip S. Bird

**Affiliations:** 1 School of Veterinary Science, Faculty of Science, The University of Queensland, Gatton, Qld, Australia

Introduction - Pa is not frequently involved in oral infections. Pa is a transitional pathogen and can be involved in opportunistic infections. Please change to indicate that. Please change throughout the article 'isolates' to 'isolate' (delete "s"). There is nothing in the introduction or discussion on the other products from bees such as honey, propolis. Why only introduce royal jelly and not these products which have anti-bacterial properties? References Pasupuleti *et al. (*2017 ^1^) and Camplin and Maddocks (2014 ^2^) would be useful additions. Was Royal jelly freeze-dried? The royal jelly came from bees, not beekeeper, and what race of bees?

 Just as an addendum, while this paper is mainly a technical paper I thought it would be of interest to readers with a few sentences on bee products as well as royal jelly. Some thing on how royal jelly is produced by bees. How and what would royal jelly be used for in treatment and what infections?

Is the work clearly and accurately presented and does it cite the current literature?

Partly

If applicable, is the statistical analysis and its interpretation appropriate?

I cannot comment. A qualified statistician is required.

Are all the source data underlying the results available to ensure full reproducibility?

Yes

Is the study design appropriate and is the work technically sound?

Yes

Are the conclusions drawn adequately supported by the results?

Yes

Are sufficient details of methods and analysis provided to allow replication by others?

Partly

Reviewer Expertise:

Oral microbiologist and beekeeper

I confirm that I have read this submission and believe that I have an appropriate level of expertise to confirm that it is of an acceptable scientific standard, however I have significant reservations, as outlined above.

## References

[1] : Honey, Propolis, and Royal Jelly: A Comprehensive Review of Their Biological Actions and Health Benefits. *Oxid Med Cell Longev* .2017;2017: 10.1155/2017/1259510 1259510 10.1155/2017/1259510 28814983PMC5549483

[2] : Manuka honey treatment of biofilms of Pseudomonas aeruginosa results in the emergence of isolates with increased honey resistance. *Ann Clin Microbiol Antimicrob* .2014;13: 10.1186/1476-0711-13-19 19 10.1186/1476-0711-13-19 24884949PMC4023514

# Response

**Published:** 2021-11-27

**Authors:** Heni Susilowati

**Affiliations:** Universitas Gadjah Mada, Indonesia

**Reviewer comment 1:**

 Introduction - Pa is not frequently involved in oral infections. Pa is a transitional pathogen and can be involved in opportunistic infections. Please change to indicate that.

**Response:**

 We have changed the first sentence of the first paragraph in the introduction as follows:

*Pseudomonas aeruginosa* ( *P. aeruginosa*) is transitional pathogen. This Gram-negative bacillus can be involved in seriously opportunistic-systemic-infections, mainly in immunocompromised patients ^1^^– ^^3^ .

**Reviewer comment 2:**

 Please change throughout the article 'isolates' to 'isolate' (delete "s").

**Response:**

 Correction was done on paragraph 4 of Introduction, paragraph 1 and 3 of Results, paragraph 4 and 8 of the Discussion section.

**Reviewer comment 3:**

 There is nothing in the introduction or discussion on the other products from bees such as honey, propolis. Why only introduce royal jelly and not these products which have anti-bacterial properties? References Pasupuleti et al. (2017) and Camplin and Maddocks (2014) would be useful additions.

**Response:**

 Additional information about honey products in the 6 ^th^ paragraph of the Discussion section, written as 4 ^th^-7 ^th^ sentences.

 The increase in pyocyanin production ……. The known bee products are honey, propolis, and royal jelly. All three have antibacterial and antibiofilm properties, in addition to other therapeutic potentials in the process of tissue healing, reproduction, and infectious diseases. (Pasupuleti et al, 2017) Manuka honey has been widely studied and proven to be antibacterial and inhibit the formation of biofilms in *P. aeruginosa*; however, bacteria derived from biofilms experienced a decrease in sensitivity to manuka honey due to the presence of variants in the bacterial population (Camplin and Maddocks, 2014) or due to subinhibitory concentrations of manuka honey. (Lu et al, 2019). Our results indicate that the sensitivity of *P. aeruginosa* bacteria to royal jelly may also vary, depending on the concentration of royal jelly exposed to the bacteria. At sub-inhibitory concentration, we found the increase of the pyocyanin production and biofilm growth. …….. of royal jelly.

**Reviewer comment 4:**

 Was Royal jelly freeze-dried?

**Response:**

 The royal jelly preparation used in this study was a fresh extract in PBS as described at the end of the first paragraph of the Methods section.

**Reviewer comment 5:**

 The royal jelly came from bees, not beekeeper, and what race of bees?

**Response:**

 The royal jelly we use is produced by *Apis mellifera* bees. We have revised it according to the reviewer's suggestion, which can be read in the 4 ^th^ sentence of the first paragraph of the Methods section.

**Reviewer comment 6:**

 Just as an addendum, while this paper is mainly a technical paper, I thought it would be of interest to readers with a few sentences on bee products as well as royal jelly. Something on how royal jelly is produced by bees. How and what would royal jelly be used for in treatment and what infections?

**Response:**

 Additional information regarding royal jelly is written at the beginning of paragraph 4 of the Introduction section as follows:

 Royal jelly is produced by hypopharyngeal and mandibular glands of worker bees. This substance is a "superfood" for the queen bee. It is widely used as a nutrient-rich supplement to overcome various health conditions such as inflammation, bacterial infection, tumor, allergy, and antioxidant and immunomodulatory effects. [Fratini et al., 2016; Pasupuleti et al., 2017]

**Response to the reviewer comments on statistical analysis:**

 We have consulted statistical analysis of the results of this study with statisticians and stated that no changes were needed.

**Additional references:**

 41. Pasupuleti VR, Sammugam L, Ramesh N, et al: Honey, propolis, and royal jelly: A comprehensive review of their biological actions and health benefits.  Oxid Med Cell Longev. 2017;2017:1259510. doi: 10.1155/2017/1259510

 42. Camplin AL and Maddocks SE: Manuka honey treatment of biofilms of *Pseudomonas aeruginosa* results in the emergence of isolates with increased honey resistance. Ann Clin Microbiol Antimicrob. 2014;13:19

 43. Lu J, Cokcetin NN, Burke CM, et al.: Honey can inhibit and eliminate biofilms produced by *Pseudomonas aeruginosa*. Sci Rep. 2019; 9(1):18160

# Peer review: Reviewer response for version 3

**DOI:** 10.5256/f1000research.77925.r95642

**Published:** 2021-10-06

**Authors:** Shizuo Kayama

**Affiliations:** 1 Antimicrobial Resistance Research Center, National Institute of Infectious Diseases, Higashimurayama, Japan

The revised manuscript has been improved compared to the previous version. I have no more comment.

Is the work clearly and accurately presented and does it cite the current literature?

Yes

If applicable, is the statistical analysis and its interpretation appropriate?

Yes

Are all the source data underlying the results available to ensure full reproducibility?

Yes

Is the study design appropriate and is the work technically sound?

Yes

Are the conclusions drawn adequately supported by the results?

Yes

Are sufficient details of methods and analysis provided to allow replication by others?

Yes

Reviewer Expertise:

bacteriology

I confirm that I have read this submission and believe that I have an appropriate level of expertise to confirm that it is of an acceptable scientific standard.

# Peer review: Reviewer response for version 3

**DOI:** 10.5256/f1000research.77925.r95641

**Published:** 2021-09-28

**Authors:** Theerthankar Das

**Affiliations:** 1 Department of Infectious Diseases and Immunology, School of Medical Science, The University of Sydney, Camperdown, NSW, Australia

I approve this manuscript.

Is the work clearly and accurately presented and does it cite the current literature?

Yes

If applicable, is the statistical analysis and its interpretation appropriate?

Partly

Are all the source data underlying the results available to ensure full reproducibility?

Partly

Is the study design appropriate and is the work technically sound?

Partly

Are the conclusions drawn adequately supported by the results?

Partly

Are sufficient details of methods and analysis provided to allow replication by others?

Yes

Reviewer Expertise:

NA

I confirm that I have read this submission and believe that I have an appropriate level of expertise to confirm that it is of an acceptable scientific standard.

# Peer review: Reviewer response for version 2

**DOI:** 10.5256/f1000research.54720.r89549

**Published:** 2021-09-07

**Authors:** Theerthankar Das

**Affiliations:** 1 Department of Infectious Diseases and Immunology, School of Medical Science, The University of Sydney, Camperdown, NSW, Australia

I approve this manuscript and recommend for indexing.

Is the work clearly and accurately presented and does it cite the current literature?

Yes

If applicable, is the statistical analysis and its interpretation appropriate?

Partly

Are all the source data underlying the results available to ensure full reproducibility?

Partly

Is the study design appropriate and is the work technically sound?

Partly

Are the conclusions drawn adequately supported by the results?

Partly

Are sufficient details of methods and analysis provided to allow replication by others?

Yes

Reviewer Expertise:

NA

I confirm that I have read this submission and believe that I have an appropriate level of expertise to confirm that it is of an acceptable scientific standard.

# Peer review: Reviewer response for version 2

**DOI:** 10.5256/f1000research.54720.r92437

**Published:** 2021-08-31

**Authors:** Shizuo Kayama

**Affiliations:** 1 Antimicrobial Resistance Research Center, National Institute of Infectious Diseases, Higashimurayama, Japan

Amly *et al.* investigated that 25% royal jelly was able to inhibit *P. aeruginosa*, but it could increase pyocyanin and biofilm production at subinhibitory concentrations. This bilateral reaction of *P. aeruginosa *against royal jelly is interesting. However, it seems that this manuscript is constructed according to mainly observation. Your estimation of the molecular mechanism brought from your observation is detail. But I am interested in the relationship between the expression of these genes and those phenotypes. I want to know the molecular mechanism more deeply. The reader of your manuscript will also want to know the gene expression related to pyocyanin, or quorum sensing, and so on. If possible, please measure the gene expression.

Is the work clearly and accurately presented and does it cite the current literature?

Yes

If applicable, is the statistical analysis and its interpretation appropriate?

Yes

Are all the source data underlying the results available to ensure full reproducibility?

Yes

Is the study design appropriate and is the work technically sound?

Yes

Are the conclusions drawn adequately supported by the results?

Yes

Are sufficient details of methods and analysis provided to allow replication by others?

Yes

Reviewer Expertise:

bacteriology

I confirm that I have read this submission and believe that I have an appropriate level of expertise to confirm that it is of an acceptable scientific standard, however I have significant reservations, as outlined above.

# Response

**Published:** 2021-09-21

**Authors:** Heni Susilowati

**Affiliations:** Universitas Gadjah Mada, Indonesia

We are very grateful for your invaluable input. We agree that an analysis of the effect of royal jelly on the expression of quorum sensing-related genes that play important roles in the regulation of biofilm formation and *P. aeruginosa* pyocyanin production is urgently needed. Accordingly, we add this discussion to the manuscript version 3, paragraph 7 of the discussion section. We have also added one related reference to the bibliography (no. 39).

# Peer review: Reviewer response for version 2

**DOI:** 10.5256/f1000research.54720.r89548

**Published:** 2021-07-22

**Authors:** Ariadna A. Djais

**Affiliations:** 1 Department of Oral Biology, Faculty of Dentistry, University of Indonesia, Jakarta, Indonesia

I confirm that I have read this submission and its revisions, and I believe that I have an appropriate level of expertise to confirm that it is of an acceptable scientific standard.

Is the work clearly and accurately presented and does it cite the current literature?

Yes

If applicable, is the statistical analysis and its interpretation appropriate?

Yes

Are all the source data underlying the results available to ensure full reproducibility?

Yes

Is the study design appropriate and is the work technically sound?

Yes

Are the conclusions drawn adequately supported by the results?

Yes

Are sufficient details of methods and analysis provided to allow replication by others?

Partly

Reviewer Expertise:

NA

I confirm that I have read this submission and believe that I have an appropriate level of expertise to confirm that it is of an acceptable scientific standard.

# Peer review: Reviewer response for version 1

**DOI:** 10.5256/f1000research.30869.r77077

**Published:** 2021-02-04

**Authors:** Theerthankar Das

**Affiliations:** 1 Department of Infectious Diseases and Immunology, School of Medical Science, The University of Sydney, Camperdown, NSW, Australia

The intention of this study is good and to enlighten the antibacterial and anti-virulence effect of royal jelly in *P. aeruginosa*.

 To be more conclusive this study needs more work:

1. Its better to use chloroform-HCl (standard and more popular) assay to quantify pyocyanin yield - its more rigorous than just taking absorbance at 690nm of pyocyanin (broad peak).
2. Biofilm (Crystal violet for biomass quantification) study needs to be done to ensure impact of royal jelly/pyocyanin on biofilm development.
3. Confocal microscopy will be of great help to show biofilm architecture.

Specific comments:

- Why solubilized royal jelly is centrifuged and supernatant is only taken? What happens with pellets (are there any pellets found after centrifugation?). If yes - then how can we know that supernatant has all royal jelly?
- At 25% royal jelly we see only 60% growth inhibition for both bacterial strains. Its not MIC - you can call it LC50 (lethal dose to reduce at least 50% growth). MIC is when there is zero or 1-2% growth only.

Is the work clearly and accurately presented and does it cite the current literature?

Yes

If applicable, is the statistical analysis and its interpretation appropriate?

Partly

Are all the source data underlying the results available to ensure full reproducibility?

Partly

Is the study design appropriate and is the work technically sound?

Partly

Are the conclusions drawn adequately supported by the results?

Partly

Are sufficient details of methods and analysis provided to allow replication by others?

Yes

Reviewer Expertise:

Biofilms, antimicrobial agents

I confirm that I have read this submission and believe that I have an appropriate level of expertise to state that I do not consider it to be of an acceptable scientific standard, for reasons outlined above.

# Response

**Published:** 2021-06-07

**Authors:** Heni Susilowati

**Affiliations:** Universitas Gadjah Mada, Indonesia

We thank you for the thorough and careful review of our manuscript. We apologize for our delay in revisions due to the various obstacles we face regarding the availability of laboratory facilities and access during the Covid-19 pandemic.

 The following are our responses to the suggestions and specific comments.

**The response** **to comment 1 (quantification of pyocyanin)**

 We measured the concentration of pyocyanin contained in the culture supernatant of *P. aeruginosa*. This condition is in accordance with the previous research conducted by Price-Whelan et al. (2007). Indeed, we only measured the absorbance value of the supernatant filtrate of the bacterial culture at a wavelength of 690 nm, but the pyocyanin concentration was then known by calculations using a formula published by previous researchers (Price-Whelan et al, 2007). The constant value of 4310 M -1cm -1 for the absorbance at 690 nm has been determined based on the research of Dietrich et al. 2006. Since our experimental conditions match the experimental conditions in both studies, both of which are published in outstanding journals, we feel it is sufficient to refer to their methods.

**The response** **to comment 2 (** **Biofilm [Crystal violet for biomass quantification] study)**

 We have conducted additional experiments to detect the association between pyocyanin production in bacteria exposed to royal jelly and biofilm formation using the static microtiter plate biofilm assay (crystal violet staining) method. To analyze the relationship between pyocyanin production and biofilm mass production, we have used *P. aeruginosa* ATCC 10145 as this strain is more responsive in producing pyocyanin when compared to the clinical isolate strains we previously use. The results showed that the detectable biofilm mass significantly increased in bacterial cultures exposed to 12.5% ​​royal jelly extract. Meanwhile, cultures treated with 25% royal jelly did not show any biofilm formation. The 6.25% royal jelly extract and the lower concentrations induced biofilm mass formation but at a lower quantity than 12.5% ​​royal jelly. This evidence suggests a relationship between royal jelly concentration, pyocyanin production, and biofilm mass formation. The subinhibitory concentration (12.5%, 6.23%) of the royal jelly extract induced more pyocyanin production and biofilm mass formation rather than the higher concentrations.

*Related changes were in the Methods, Results, and Conclusion of the Abstract, paragraph 6 of the Methods, paragraph 5 of Results, Figure 4, and line 5 of paragraph 6 on the Discussion section in the manuscript.*

**The response** **to comment 3 (microscopy of biofilm architecture)**

 In order to complement the data regarding the effect of royal jelly on the mass formation of biofilms, we have carried out observations of the microscopic architecture of *P. aeruginosa* ATCC 10145 biofilms using scanning electron microscopy, since we could not access the confocal microscopy method at our university. We chose secondary electron (SE) mode rather than a backscattered electron (BSE) to investigate biofilm in a three-dimensional perspective. The results of observations on representative samples showed that in the culture exposed to 25% royal jelly extract there was no biofilm mass deposition, while the group exposed to 12.5% royal jelly showed a larger density of biofilm than the 6.25% royal jelly group. Chlorhexidine 0.2% control showed total inhibition of biofilm mass formation.

 Methods, results, discussion, and related references have been added to the manuscript on Methods, Results, and Conclusion in the Abstract, paragraph 7 of the Methods section, paragraph 6-7 of the Results section, and line 5 in paragraph 6 of the Discussion section.

**Specific comments:**

**1. Why solubilized royal jelly is centrifuged and supernatant is only taken?**

**Answer:** The extraction method using phosphate buffer saline (Hu et al, 2019) was chosen in order to dissolve major royal jelly protein 1 (MRJP1), the active components that are antibacterial or anti-adhesion. It is known that MRJP1 is a hydrosoluble protein dissolved in PBS, which is higher in supernatants than in pellets (Gismondi et al., 2017).

**What happens with pellets (are there any pellets found after centrifugation?). If yes - then how can we know that supernatant has all royal jelly?**

 After centrifugation, there were indeed pellets, and it has been found that the pellets contain more liposoluble proteins than supernatants. To ensure that the compounds contained in the PBS royal jelly extract supernatant, further research is needed; However, from the research of Furusawa et al. (2016), it was found that MRJP1 was contained in the supernatant of PBS royal jelly extract in quite a large amount, reaching 27.6%.

 The active components that are antibacterial or anti-adhesion in the supernatant of royal jelly extract in this study cannot be explained with certainty, but the royal jelly used in this study was thought to mainly contain MRJP1.

 We add this explanation to paragraph 2 of the Discussion section. Additional sources of literature have been written in the reference list numbers 37, 38, and 39.

**2.** **At 25% royal jelly, we see only 60% growth inhibition for both bacterial strains. It is not MIC - you can call it LC50 (lethal dose to reduce at least 50% growth). MIC is when there is zero or 1-2% growth only.**

**Answer:** We agree with the reviewer's comments regarding the MIC score. In clinical isolates, 25% royal jelly extract caused 60% death in the bacterial population; meanwhile, in the standard strain ( *P. aeruginosa* ATCC 10145), this concentration caused growth inhibition of 85%.

 We have revised the first sentence in Methods of Abstract and two last sentences in the Results section.

# Response

**Published:** 2021-06-11

**Authors:** Heni Susilowati

**Affiliations:** Universitas Gadjah Mada, Indonesia

We thank you for the thorough and careful review of our manuscript. 

 The following are our responses to the suggestions and specific comments.

**The response** **to comment 1 (quantification of pyocyanin)**

 We measured the concentration of pyocyanin contained in the culture supernatant of *P. aeruginosa*. This condition is in accordance with the previous research conducted by Price-Whelan et al. (2007). Indeed, we only measured the absorbance value of the supernatant filtrate of the bacterial culture at a wavelength of 690 nm, but the pyocyanin concentration was then known by calculations using a formula published by previous researchers (Price-Whelan et al, 2007). The constant value of 4310 M -1cm -1 for the absorbance at 690 nm has been determined based on the research of Dietrich et al. 2006. Since our experimental conditions match the experimental conditions in both studies, both of which are published in outstanding journals, we feel it is sufficient to refer to their methods.

**The response to comment 2 (Biofilm [Crystal violet for biomass quantification] study)**

 We have conducted additional experiments to detect the association between pyocyanin production in bacteria exposed to royal jelly and biofilm formation using the static microtiter plate biofilm assay (crystal violet staining) method. To analyze the relationship between pyocyanin production and biofilm mass production, we have used *P. aeruginosa* ATCC 10145 as this strain is more responsive in producing pyocyanin when compared to the clinical isolate strains we previously use. The results showed that the detectable biofilm mass significantly increased in bacterial cultures exposed to 12.5% royal jelly extract. Meanwhile, cultures treated with 25% royal jelly did not show any biofilm formation. The 6.25% royal jelly extract and the lower concentrations induced biofilm mass formation but at a lower quantity than 12.5% royal jelly. This evidence suggests a relationship between royal jelly concentration, pyocyanin production, and biofilm mass formation. The subinhibitory concentration (12.5%, 6.23%) of the royal jelly extract induced more pyocyanin production and biofilm mass formation rather than the higher concentrations.

*Related changes were in the Methods, Results, and Conclusion of the Abstract, paragraph 6 of the Methods, paragraph 5 of Results, Figure 4, and line 5 of paragraph 6 on the Discussion section in the manuscript.*

**The response** **to comment 3 (microscopy of biofilm architecture)**

 In order to complement the data regarding the effect of royal jelly on the mass formation of biofilms, we have carried out observations of the microscopic architecture of *P. aeruginosa* ATCC 10145 biofilms using scanning electron microscopy, since we could not access the confocal microscopy method at our university. We chose secondary electron (SE) mode rather than a backscattered electron (BSE) to investigate biofilm in a three-dimensional perspective. The results of observations on representative samples showed that in the culture exposed to 25% royal jelly extract there was no biofilm mass deposition, while the group exposed to 12.5% royal jelly showed a larger density of biofilm than the 6.25% royal jelly group. Chlorhexidine 0.2% control showed total inhibition of biofilm mass formation.

*Methods, results, discussion, and related references have been added to the manuscript on Methods, Results, and Conclusion in the Abstract, paragraph 7 of the Methods section, paragraph 6-7 of the Results section, and line 5 in paragraph 6 of the Discussion section.*

**Specific comments**

**a. Why solubilized royal jelly is centrifuged and supernatant is only taken?**

**Answer:** The extraction method using phosphate buffer saline (Hu et al, 2019) was chosen in order to dissolve major royal jelly protein 1 (MRJP1), the active components that are antibacterial or anti-adhesion. It is known that MRJP1 is a hydrosoluble protein dissolved in PBS, which is higher in supernatants than in pellets (Gismondi et al., 2017).

**What happens with pellets (are there any pellets found after centrifugation?). If yes - then how can we know that supernatant has all royal jelly?**

**Answer:** After centrifugation, there were indeed pellets, and it has been found that the pellets contain more liposoluble proteins than supernatants. To ensure that the compounds contained in the PBS royal jelly extract supernatant, further research is needed; However, from the research of Furusawa et al. (2016), it was found that MRJP1 was contained in the supernatant of PBS royal jelly extract in quite a large amount, reaching 27.6%.

 The active components that are antibacterial or anti-adhesion in the supernatant of royal jelly extract in this study cannot be explained with certainty, but the royal jelly used in this study was thought to mainly contain MRJP1.

*We add this explanation to paragraph 2 of the Discussion section. Additional sources of literature have been written in the reference list numbers 37, 38, and 39.*

**b. At 25% royal jelly, we see only 60% growth inhibition for both bacterial strains. It is not MIC - you can call it LC50 (lethal dose to reduce at least 50% growth). MIC is when there is zero or 1-2% growth only.**

**Answer:** We agree with the reviewer's comments regarding the MIC score. In clinical isolates, 25% royal jelly extract caused 60% death in the bacterial population; meanwhile, in the standard strain ( *P. aeruginosa* ATCC 10145), this concentration caused growth inhibition of 85%.

*We have revised the first sentence in Methods of Abstract and two last sentences in the Results section.*

# Peer review: Reviewer response for version 1

**DOI:** 10.5256/f1000research.30869.r77076

**Published:** 2021-02-01

**Authors:** Ariadna A. Djais

**Affiliations:** 1 Department of Oral Biology, Faculty of Dentistry, University of Indonesia, Jakarta, Indonesia

This study examined royal jelly and *Apis mellifera *bees as a base ingredient. These ingredients are antibacterial with pycocyanin as a pigment by the bacterium *Psedomonas aeroginosa. *The *P. aeroginosa *was selected because these bacteria are known as multidrug-resistant bacterium. This research is interesting and specific. In this study, the data was analyzed as an indicator of the difference in the contrast of the green pigment color, which was carried out by measuring at a wavelength of 690 nm.

 In my opinion, this research needs to add more information regarding the methods chapters:

1. It is necessary to add information about the stage of royal jelly making as the test material.
2. It is necessary to add information about the bacterium identification technique which used by the researcher when confirming clinical isolates as *P. aeroginosa.*
3. Number of **replication of independent experiment conducted by the researcher.

Is the work clearly and accurately presented and does it cite the current literature?

Yes

If applicable, is the statistical analysis and its interpretation appropriate?

Yes

Are all the source data underlying the results available to ensure full reproducibility?

Yes

Is the study design appropriate and is the work technically sound?

Yes

Are the conclusions drawn adequately supported by the results?

Yes

Are sufficient details of methods and analysis provided to allow replication by others?

Partly

Reviewer Expertise:

NA

I confirm that I have read this submission and believe that I have an appropriate level of expertise to confirm that it is of an acceptable scientific standard, however I have significant reservations, as outlined above.

# Response

**Published:** 2021-02-14

**Authors:** Heni Susilowati

**Affiliations:** Universitas Gadjah Mada, Indonesia

Thank you for providing a review and suggestions for improving our manuscript. Below are our revisions to the manuscript according to the review comments.

**1. Response to comments 1:**

 We explain the stages of preparing royal jelly on the manuscript, written in the Methods section (page 3) as follows:

 Royal jelly used in this study was obtained from Nusukan, Surakarta, Central Java, Indonesia and harvested in May-October 2019. This product was obtained from the beekeeper Apis mellifera who lives in the randu trees ( *Ceiba pentandra*) and sono wood ( *Dalbergia latifolia*). Apis mellifera bee species have been identified through previous research19. Royal jelly 5.5 grams was dissolved in 10 ml of cold phosphate buffered saline (PBS), then homogenized using a magnetic stirrer (24 hours, 4 ^o^C). The royal jelly solution was centrifuged (12,000 g, 45 minutes, 4°C), then the supernatant is taken and checked the pH. The results of pH measurements with a pH meter showed a pH of 3.79. Furthermore, the supernatant was filtered using 0.45 µm Millipore to produce 55% royal jelly.

**2. Response to comment 2:**

 We write an explanation of how to identify bacteria in the Methods section (page 3), which is as follows:

 Identification of the clinical isolate was carried out using API 2ONE (bioMérieux, Inc).

**3. Response to comment 3:**

 Each experiment was carried out with 6 replications.

 We write the revision on page 3 of the Methods section, on bacterial viability tests and pyocyanin detection.

# Response

**Published:** 2021-06-11

**Authors:** Heni Susilowati

**Affiliations:** Universitas Gadjah Mada, Indonesia

Thank you for providing a review and suggestions for improving our manuscript.

 Below are our revisions to the manuscript according to the review comments.

 1. **The response to comment 1**

 We explain the stages of preparing royal jelly on the manuscript, written in *line 2-6 of paragraph 2 of the Methods* section as follows:

 Royal jelly used in this study was obtained from Nusukan, Surakarta, Central Java, Indonesia and harvested in May-October 2019. This product was obtained from the beekeeper *Apis mellifera* who lives in the randu trees ( *Ceiba pentandra*) and sono wood ( *Dalbergia latifolia*). *Apis mellifera* bee species have been identified through previous research (Hartono et al., 2019). Royal jelly 5.5 grams was dissolved in 10 ml of cold phosphate buffered saline (PBS), then homogenized using a magnetic stirrer (24 hours, 4 ^o^C). The royal jelly solution was centrifuged (12,000 g, 45 minutes, 4°C), then the supernatant was taken and checked for the pH. The results of pH measurements using a pH meter showed a pH of 3.79. Furthermore, the supernatant was filtered using 0.45 µm Millipore to produce 55% royal jelly.

**2. The response to comment 2**

 We have written an explanation of how to identify bacteria in *line 5 of paragraph 3 the Methods section,* which is as follows:

 Identification of the clinical isolate was carried out using API 2ONE (BioMérieux, Inc).

**3. Response** **to comment 3**

 The bacterial viability assay experiment was carried out in quadruplicate, meanwhile, the pyocyanin experiments were performed in triplicate.

*We write the revision in the last line of related paragraphs in the Methods section.*
